# Mapping the *PIK3CA*-related overgrowth spectrum (PROS) patient and caregiver journey using a patient-centered approach

**DOI:** 10.1186/s13023-022-02338-1

**Published:** 2022-05-07

**Authors:** Lara Rodríguez-Laguna, Kristen Davis, Mellenee Finger, Dawn Aubel, Robin Vlamis, Craig Johnson

**Affiliations:** 1 Vascular Malformations Section, Institute of Medical and Molecular Genetics, INGEMM-IdiPAZ, La Paz University Hospital, Paseo de La Castellana, 261, 28046 Madrid, Spain; 2 CLOVES Syndrome Community, West Kennebunk, ME USA; 3 K-T Support Group, Milford, OH USA; 4 Novartis Pharmaceuticals Corporation, East Hanover, NJ USA; 5 Solstice HealthCommunications, Far Hills, NJ USA; 6 Interventional Radiology, Nemours Children’s Hospital, Orlando, FL USA

**Keywords:** *PIK3CA*, PI3K, PROS, Vascular malformation

## Abstract

**Background:**

PROS disorders are driven by somatic, gain-of-function mutations in *PIK3CA* that result in hyperactivation of the phosphatidylinositol-3-kinase (PI3K) signaling pathway. PROS encompasses a broad spectrum of overlapping phenotypes (including overgrowth and vascular malformations) that vary significantly in their severity; every case is unique, leading to different, complex experiences. Here, we aim to describe the PROS experience from the patients’ and caregivers’ points of view, from onset to diagnosis to treatment and support.

**Results:**

The PROS patient journey was developed using a literature review, an ethnography study, health care professional (HCP) research, and social listening. It was then validated with patients, caregivers, and patient advocates. Physician research included 94 PROS centers and other vascular anomaly centers throughout the United States and Europe. Ethnographic research included 24 patients, caregivers, and/or advocates; selected data from 223 patients were reviewed. Key priority areas of need were identified, along with barriers to and potential enablers of quality care. Visual mapping of the PROS patient and family journey was developed to identify key personal health and system issues, and opportunities for improvements throughout patients’ lifespans. Maps were also developed for 3 specific conditions: Klippel–Trénaunay syndrome (K–T); congenital lipomatous overgrowth, vascular malformations, epidermal nevi, scoliosis/skeletal and spinal anomalies (CLOVES) syndrome; and megalencephaly-capillary malformation syndrome (M-CM). Overall, most patients with PROS conditions and their families struggle with a long path to diagnosis, access to genetic testing, and finding qualified specialists. Following diagnosis, patients and families are frequently challenged with major medical events, comorbidities, unpredictability, frequent hospitalization, impact on school and work, the need for multidisciplinary care, unwanted attention, adverse impact on mental and emotional health, and financial pressures. Lack of effective pain management emerged as a substantial issue. Challenges and barriers to quality care shift throughout patients’ lifespans; transition from pediatric to adult care can be especially difficult.

**Conclusions:**

This patient journey in PROS was created in collaboration with patients, caregivers, and advocates as key partners. This novel methodology, which could be applied elsewhere, can more accurately identify areas of unmet need, barriers to care, education topics, and assist HCPs to understand the patient and family perspective.

**Supplementary Information:**

The online version contains supplementary material available at 10.1186/s13023-022-02338-1.

## Background

*PIK3CA*-related overgrowth spectrum (PROS) is a group of rare disorders driven by somatic, gain-of-function mutations in phosphatidylinositol-4,5-bisphosphate 3-kinase catalytic subunit alpha (*PIK3CA*) [1, 2]. Mutations are associated with hyperactivation of the phosphatidylinositol-3-kinase (PI3K) signaling pathway and downstream effectors such as protein kinase B (AKT) and mammalian target of rapamycin (mTOR), leading to vascular malformations and abnormal tissue growth [1–3]. PROS encompasses a broad spectrum of overlapping phenotypes, including overgrowth and vascular malformations, that also vary significantly in their severity [1, 4]. PROS, which includes at least 13 distinct disorders, composes one category of *PIK3CA*-related disorders that also includes *PIK3CA*-related vascular malformations as well as *PIK3CA*-related nonvascular lesions [5].

Manifestations associated with these conditions can be severe and affect multiple elements of patients’ lives (physical limitations to activities of daily living, social stigma, mental health, repeated surgeries, trips to the emergency room); management requires a multidisciplinary approach due to the wide scope of potential comorbidities [6–8]. Among PROS disorders, megalencephaly-capillary malformation (M-CM or MCAP) syndrome, Klippel–Trénaunay syndrome (K–T or KTS), and congenital lipomatous overgrowth, vascular malformations, epidermal nevi, scoliosis/skeletal and spinal anomalies (CLOVES) syndrome are particularly challenging for patients because many tissues and body parts may be affected (Table 1) [3, 9–11].

**Table 1:** Clinical characteristics associated with CLOVES syndrome, K–T syndrome, and M-CM

PROS condition	Clinical characteristics
CLOVES [3, 9]	Overgrowth (lipomatous, spinal/paraspinal, bony, limb and digit); cutaneous and vascular malformations (lymphatic, venous, capillary ± arteriovenous malformations); varicose embryonic veins and blood clots; musculoskeletal abnormalities; visceral abnormalities; neurologic abnormalities
K–T [3, 10, 11]	Vascular malformations (capillary, venous, ± lymphatic malformations) or varicose embryonic veins and blood clots; hypertrophy of bone and soft tissue (features are typically isolated to the pelvis and a lower extremity, but upper extremities and other organs can be involved)
M-CM [3, 9]	Segmental overgrowth of brain tissue (megalencephaly or hemimegalencephaly); cutaneous capillary malformations with focal or generalized somatic overgrowth; digital anomalies; tone abnormalities; mild to severe intellectual disability may be present

CLOVES, congenital lipomatous overgrowth, vascular malformations, epidermal nevi, scoliosis/skeletal and spinal anomalies; K–T, Klippel–Trénaunay; M-CM, megalencephaly-capillary malformation

Due in part to their rarity, research in PROS-related disorders is limited and there is a lack of awareness and education among not only health care professionals (HCPs) but among patients, their caregivers, and their families. Every case is unique, leading to different, complex experiences for all involved. Patients, caregivers, and families experience long-term challenges with ongoing care over the course of the disease, including finding HCPs who have experience treating patients with PROS conditions, and access to appropriate specialists with knowledge of emerging systemic treatments [12–14].

Here, we present the results of a novel approach to the study of the patient/caregiver experience journey across PROS disorders from onset to diagnosis, treatment, and support. The aim of this research is to raise awareness of these rare conditions, increase understanding of the patient and caregiver experiences, and improve the care that patients receive.

## Methods

### Data collection

Literature searches were conducted using PubMed with search terms related to PROS and PROS disorders to identify peer-reviewed literature published from 2007 to 2020. Information was gathered from advocacy and institution websites including those of Boston Children’s Hospital, the CLOVES Syndrome Community, the International Society for the Study of Vascular Anomalies (ISSVA), K-T Support Group, Lymphangiomatosis and Gorham-Stout Disease Alliance (LGD Alliance), M-CM Network, Mayo Clinic, the National Institutes of Health (NIH), the National Organization for Rare Disorders (NORD), Project FAVA, PROSSpectrum, and WonderFIL Smiles. Clinical practice guidelines were reviewed where available.

Ethnography research was conducted with a group of 10 adult patients and 14 caregivers of pediatric patients; all participants were recruited through patient advocacy groups. CLOVES syndrome, K–T syndrome, and M-CM were represented by 8 respondents each. Data were collected using telephone interviews, discussions conducted in-home and online, and personal journals. Research involving HCPs was conducted with physicians from the United States (US), Germany, and France. Qualitative research was intended to determine drivers of patient identification, current treatment approaches and triggers for treatment, and key challenges in patient flow and management (US, n = 25; Germany, n = 8). Quantitative research was intended to determine rates of diagnosis and testing, and most common treatments, sites of care, referral patterns, and treating specialties (US, n = 50; Germany, n = 21; France, n = 16). Selected, anonymized data from patients with PROS conditions (US, n = 105; Germany, n = 49; France, n = 69) were collected from treating physicians and nurse practitioners to quantify diagnosis, disease classification, treatment initiation, and treatment history. Trends and concerns among patients, caregivers, and families were also monitored by social media listening (Twitter, Instagram, Facebook, Reddit, discussion forums, and blogs).

This work was not reviewed by an institutional review board as this study was focused on information collection and did not involve medical interventions.

### Analysis of findings

Core insights were identified with respect to symptoms and disease experience, disease management and care, emotional and social impact of PROS, impact of PROS on the family, and sources of education and support. Patient journeys were mapped and subsequently revised and validated based on feedback obtained from advisory boards, telephone interviews, and 10 leaders from 9 PROS advocacy groups (M-CM Network, Project FAVA, LGD Alliance, Hevas, WonderFIL Smiles, the CLOVES Syndrome Community, Asociación M-CM España, K-T Support Group, and Associazione Italiana Macrodattilia e PROS).

## Results

**Fig. 1:**
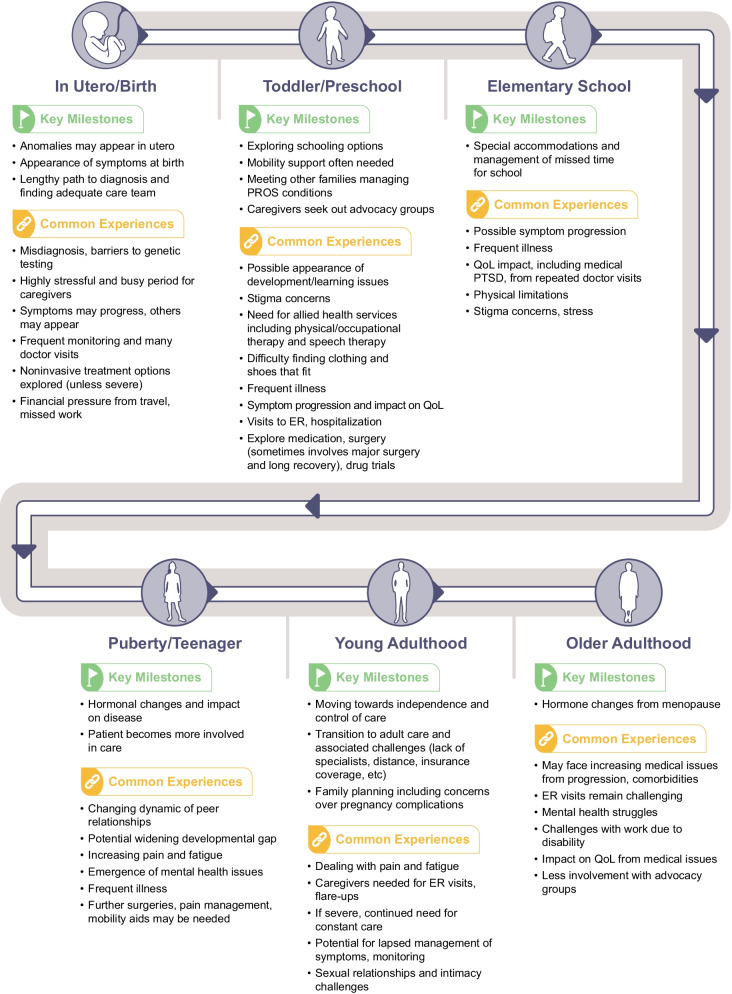
The PROS Journey. This figure depicts milestones and common experiences for patients and caregivers across PROS disorders, although individual experiences will differ depending on the disorder and severity of disease. Further details on the PROS journey and specific journeys for K-T, CLOVES, and M-CM are provided in Additional file 1. CLOVES, congenital lipomatous overgrowth, vascular malformations, epidermal nevi, scoliosis/skeletal and spinal anomalies syndrome; ER, emergency room; K–T, Klippel–Trénaunay syndrome; M-CM, megalencephaly-capillary malformation syndrome; *PIK3CA,* phosphatidylinositol-4,5-bisphosphate 3-kinase catalytic subunit alpha; PROS, *PIK3CA*-related overgrowth spectrum; PTSD, post-traumatic stress disorder, QoL, quality of life

The PROS patient and caregiver journey was mapped from the time of somatic mutation in utero through to adulthood (Fig. 1 and Additional file 1). Disease characteristics and associated challenges to patients, caregivers, and families were noted for K–T, CLOVES, and M-CM (as well as across PROS disorders) at 6 key phases of development: birth, toddler/preschool age, elementary school age, puberty/teenage years, young adulthood, and older adulthood (Table 2).

**Table 2:** Key elements and challenges at different stages of life across PROS disorders

Stage of life	Key elements and challenges			
	Across PROS	Specific to K–T	Specific to CLOVES	Specific to M-CM
Birth to toddler	Appearance of symptoms: vascular anomalies, port-wine stain, soft/bony tissue hypertrophy, asymmetrical overgrowth, enlarged head, macrodactyly Challenge finding qualified specialists	Appearance of symptoms: Capillary malformations present as port-wine stains Venous malformations present as varicose veins Lymphatic malformations present as lymphedema Vast majority of extremity hypertrophy occurs in the leg	Appearance of symptoms: abnormal extremities, fatty truncal mass, vascular anomalies, skin abnormalities	Appearance of symptoms: capillary malformations, “purple body,” webbed/extra digits, asymmetrical overgrowth, enlarged head, midline facial birthmark, low muscle tone
Toddler/preschool age	Stigma concerns Support devices for mobility Exploring appropriate schooling options and support Frequent illnesses	Support devices for mobility Special clothing supplier/tailoring Stigma concerns can arise due to greater interaction with other children Begin thinking about schooling options Watch for trauma/infections	Developmental/learning issues may begin to appear Stigma concerns Support devices for mobility Exploring appropriate schooling options and support Frequent illnesses	Developmental delays may appear or become more apparent Support devices for mobility, communication Exploring appropriate schooling options and support Frequent illnesses
Elementary school	Special accommodations at school Missed school/work due to appointments and surgeries Physical limitations Stress from looking different, being stared at Frequent illnesses	Work with school about special accommodations for physical limitations (physical education restrictions, mobility issues, flares) Missed school time due to doctor’s appointments and surgeries Stress/anxiety from looking different (clothing issues/compression garments) Flares (bleeding, infection) Watch for signs of scoliosis	Special accommodations at school Missed school/work due to appointments and surgeries Physical limitations Stress from looking different, being stared at Frequent illnesses	Special accommodations at school as developmental delays become more obvious Missed school/work due to appointments and frequent illnesses Physical and cognitive limitations Stress on family unit, restrictions on activities Children are often socially active, happy, but some may have behavioral disorders, such as autism or anxiety
Puberty/teenage years	Hormonal changes with puberty can impact disease course Developmental gap can widen Pain and fatigue can become an increasing issue Relationships with peers can change–bullying may occur and may start to deal with intimacy/sexual health issues Anger and depression can emerge Frequent illnesses	Hormonal changes with puberty can impact disease course, causing even “mild” cases to become more complex Puberty brings an increase in clotting issues (DVT, PE, thrombophlebitis), as well as painful menstrual periods and/or weeping of lymphatic fluid that can cause stigma and shame Flares also can become more frequent and pain can become worse Watch for signs of scoliosis Relationships with peers can change along with a period of anger because of stigma and limitations on activities Teens start to deal with sexual health issues, including intimacy and birth control concerns	Hormonal changes with puberty can impact disease course Gap in development can widen; less support provided at school Pain and fatigue can become an increasing issue Girls may have heavy, painful menstruation Relationships with peers can change, bullying can occur and may start to deal with intimacy/sexual health issues Frequent illnesses Unable to participate in many activities	Hormonal changes with puberty can impact disease course Developmental gap can widen Special education services at school May have difficulty with peers Frequent illnesses, low energy
Young adulthood	Move toward independent living Transitioning to an adult care team Struggle to educate themselves on their condition Navigating intimate relationships and dealing with sexual health issues and genito-urological complications Discussions on family planning Patients with more severe physical/mental challenges remain at home and rely heavily on caregivers	Move toward independent living Transitioning to an adult care team Struggle to educate themselves on their condition Patients with more severe physical challenges remain at home and rely heavily on caregivers Continue dealing with sexual health issues, including birth control and genito-urological complications Discussions on family planning	Move toward independent living Transitioning to an adult care team Taking responsibility for own care Navigating intimate relationships and dealing with sexual health issues and genito-urological complications Discussions on family planning Patients with more severe physical/mental challenges remain at home and rely heavily on caregivers	Mildly affected patients may move toward independent living Transitioning to an adult care team May start to take responsibility for own care Patients with more severe physical/mental challenges remain at home and rely heavily on caregivers Parents may need to make decisions about guardianship
Older adulthood	Increasing medical issues due to disease progression and comorbidities Pain and fatigue leading to depression Therapist/psychologist support Potential disability, difficulty working full time	Increasing medical issues due to progression and comorbidities Pain and fatigue leading to depression Difficulty maintaining relationships Inability to work full time due to fatigue, frequent infections, hospitalizations Extensive planning needed for many activities	Increasing medical issues due to progression and comorbidities Pain and fatigue leading to depression Therapist/psychologist support Potential disability, difficulty working full time Reliance on mobility devices/support	*Data were not available for this age group*

CLOVES, congenital lipomatous overgrowth, vascular malformations, epidermal nevi, scoliosis/skeletal and spinal anomalies; DVT, deep venous thrombosis; K–T, Klippel–Trénaunay; M-CM, megalencephaly-capillary malformation; PE, pulmonary embolism; PROS, *PIK3CA*-related overgrowth spectrum

### Path to diagnosis

Some PROS conditions may be apparent very early in development. Routine ultrasound performed during pregnancy may reveal a potential issue with the fetus, such as an enlarged head, enlarged or asymmetrical limbs, extra fluid, or extra/fused digits. These findings may lead to additional tests and scans, and parents may feel nervous and unsure about what to do to help their child. When an anomaly is visible at birth, parents are typically worried and anxious, and may realize their lives and their child’s life will not be what they envisioned. Other PROS conditions, such as fibroadipose vascular anomaly (FAVA) and complex lymphatic anomaly (CLA), tend to appear later; in this case, parents may be unprepared and feel overwhelmed at the thought of their child having a rare, complex medical condition.

Due to the rarity of PROS, patients and their families may struggle for years to obtain a correct diagnosis. They are often referred to a succession of different specialists depending on the type of anomaly, thereby delaying appropriate treatment. After symptoms appear, patients are most likely to be seen by family pediatricians (US, 80%; Germany, 67%; France, 88%), general practitioners (US; 50%, Germany; 38%, France, 50%), or dermatologists (US, 38%; Germany, 57%; France, 44%). In Germany, patients are often quickly referred to a geneticist at a rare disease reference center if a genetic disorder is suspected. In the US, a majority of patients with PROS conditions (69%) had disease onset at birth or in utero; time to diagnosis of PROS was slightly longer in patients with severe disease vs nonsevere disease (11.6 months vs 8.1 months, respectively). Our findings in the US revealed that patients with severe disease were much more likely to have received an incorrect or incomplete diagnosis compared with nonsevere disease (22% vs 3%, respectively); clinical diagnosis is complicated by the variability and overlapping phenotypes of PROS disorders [1]. Many doctors are unfamiliar with PROS conditions and may do extensive testing and biopsies that could be harmful. Diagnoses may vary widely depending on the experience of the HCP team, and patients could be diagnosed with one PROS condition only to be rediagnosed with a different PROS condition many years later.

A major turning point for parents can be finding a qualified specialist to treat their child. Many states in the US do not have specialized vascular anomaly centers or children’s hospitals with expertise for management of PROS conditions; multidisciplinary centers with experienced physicians tend to be located in major population centers, with regional clusters on the East and West coasts and in the Midwest.

Overall, the factors to faster diagnosis were knowledgeable doctors, birth in an academic center, living in a nonrural area, extensive research by families, and early contact with advocacy groups. Receiving an official diagnosis provides patients and families relief, hope, affirmation, treatment options, and a monitoring protocol. A panel discussion in which the authors discuss the path to diagnosis of PROS is provided in Additional file 2.

### Genetic testing

Confirmation of a *PIK3CA* mutation can provide some relief to patients who were struggling to obtain a correct diagnosis because many patients continue to be misdiagnosed due to overlapping clinical phenotypes. A genetic diagnosis is key to directing the management of patients and may offer an opportunity for access to emerging targeted therapies. However, many patients and families reported substantial barriers. Testing can be a lengthy, expensive, and painful process, and due to the mosaic nature of *PIK3CA*-related disorders and challenges with tissue biopsy collection, it may not always be possible to confirm the presence of a *PIK3CA* mutation [1]. Access to testing outside major specialty centers is limited. HCPs inexperienced with PROS may be unfamiliar with the type of testing needed, leading to frustration on the part of parents. In addition, there is lack of consensus on the genetic diagnostic protocol with respect to optimal sequencing and validation techniques, and standards and guidelines for the interpretation of somatic *PIK3CA* variants outside the oncology setting. Because of the lack of good educational resources for families, many do not understand the genetic testing process or the reason for testing and lack knowledge regarding mosaic mutations and the need for multiple tissue samples. Parents also do not always realize that a negative test does not necessarily signify absence of a mutation in their child, only that the sample did not contain a detectable mutation, or that the genetic testing technique used is not suitable. Appropriate genetic counseling to explain these issues, as well as the genetic results, is especially important. Depending on where the affected tissue is located, biopsy procedures could be simple and painless (eg, punch biopsy in skin) or painful and invasive. Some additional challenges may arise for non-collaborative children (who may require sedation) or those with certain risk factors. For those reasons a biopsy is often scheduled to coincide with another necessary surgery or procedure, or stored formalin-fixed paraffin embedded (FFPE) tissue samples are used. Buccal swab testing is a less-invasive first option for patients with facial or head involvement, such as those with M-CM or facial infiltrating lipomatosis (FIL). Moreover, the higher mutational burden in patients with M-CM may permit detection by this method [15]. However, based on the authors’ experience, buccal swab testing is not always performed due to lack of knowledge of the disorder, unfamiliarity with this technique, lack of access to laboratories offering this test, physician preference for tissue samples, and financial constraints; separate consent may be required and insurance companies may not reimburse testing due to lack of an approved systemic therapy. Emerging techniques, such as the use of cell-free DNA, could play a role in genetic diagnosis of patients with PROS, but in the authors’ experience, identification of variants with low mosaicism remains a challenge in such samples.

### Disease experience

Many patients and families struggle to maintain a normal routine because of uncertainty regarding the course of the disease. Patients with purely cosmetic issues or isolated, localized disease in noncritical areas may experience minimal impact on activities of daily living. Conversely, patients with substantial overgrowth, malformations close to major organs, as well as patients with developmental disorders, may be severely affected and experience a large functional impact on daily activities. Among patients with CLOVES syndrome, K–T syndrome, fibroadipose hyperplasia (FH/FAO), and isolated lymphatic malformation (ILM), frequent infections may develop into cellulitis or sepsis, resulting in hospitalization and intravenous antibiotics; recovery can be long, painful, and difficult. Seizures may occur among patients with M-CM, FIL, and dysplastic megalencephaly (DMEG), and fear of onset of seizures is very worrisome for families. Other findings on the PROS disease experience are described in Table 2.

Onset of puberty can lead to increased growth and pain in some patients. Teenagers may develop anxiety and depression if their medical condition limits social and physical activities with family and friends. Patients with PROS conditions are often advised to avoid pregnancy due to the risk from worsening vascular or lymphatic malformations; however, research has shown that some women with K–T syndrome can have normal pregnancies with proper precautions and medical management [16–18]. Patients often experience side effects from medications, or comorbidities such as diabetes or gastrointestinal problems. Patients with FIL, CLOVES syndrome, ILM, and M-CM often have craniofacial malformations that cannot be concealed and may lead to decreased self-esteem. Such highly visible malformations lead to additional social stigma and unwanted attention. Patients with M-CM face additional challenges such as developmental delays and cognitive issues. Caregivers and families also contend with a number of challenges, which are described in Table 3.

**Table 3:** Challenges faced by caregivers and families

Stage of life	Challenges for caregivers and families	
	Across PROS	Specific challenges for M-CM
Birth to toddler	Primary caregiver (usually mother) needs to work full time on childcare, monitoring medical issues, arranging appointments, and research Caregiver becomes “case manager” balancing HCP communication, research, symptom management, health insurance claims	Brain overgrowth occurs most dramatically during the first few years, leading to many health challenges and the need for frequent monitoring and possibly surgeries Children may have low muscle tone, frequent infections, and delayed milestones
Elementary school	Caregiver has to work with school, explain issues, navigate missed schooldays	Severely affected patients require constant care and assistance with feeding, toileting, dressing, etc Need to prepare for and manage seizures Caregivers may seek out community-based resources and mobility devices (eg, adaptive strollers)
Puberty/teenager	Patient becomes more involved but still dependent on caregiver for research and advocacy group involvement	Family is often still involved in care—especially in severe cases Behavior issues may arise, putting additional stress on caregivers
Adulthood	Patient manages care but caregivers may stay involved for emergencies	Challenges with guardianship and consent after age 18 Patients still reliant on help from family with navigating medical care and health issues

HCP, health care professional; M-CM, megalencephaly-capillary malformation; PROS, *PIK3CA*-related overgrowth spectrum

### Management and treatment of PROS

When learning of the available treatment options, the reaction from patients and families may be mixed. Some may feel hopeful and encouraged, whereas others may be frustrated by the lack of curative therapy for their condition. Major surgery is often required to correct overgrowth, and multiple surgeries over the patient’s lifespan may be required. If amputation is recommended for their child, parents are faced with an extremely difficult decision and often question whether they are making the right choice. A general lack of outcomes research in PROS means patient experiences are not being effectively captured. Patients, families, and care teams may be required to make treatment decisions without having a thorough understanding of the efficacy of available treatment options. Systemic treatments that target the underlying disease pathways (PI3Kα, AKT, mTOR) are under clinical development [5]. Patients with PROS and their families are often aware of these potential treatments, but at present access is limited to clinical trials or compassionate use programs. Patients and families may struggle to receive a referral to a clinical trial or may be unable to travel to a specialized center where trials are conducted.

Support from allied health services plays an important role for families; physical/occupational therapy (PT/OT), feeding therapy (FT), and speech therapy (ST) are especially helpful in the early years. However, these services are often engaged separately from the primary treating HCP’s care and are not always well coordinated. A panel discussion in which the authors discuss treatment decision-making is provided in Additional file 3.

As patients enter adulthood, they and their families face a difficult transition from pediatric to adult care, particularly for those with developmental delays. Infections and other major health issues may impact plans to live independently. Pain and fatigue are significant challenges for many patients with PROS conditions, particularly for adults. Pain associated with these conditions is not well understood and is often poorly managed. Other challenges associated with management of PROS conditions are described in Tables 2 and 3.

Early in the patient’s life, clinicians first try to manage their conditions conservatively with noninvasive therapy and devices to maintain quality of life. Conservative therapies also play a role in management of certain manifestations of PROS throughout the patient’s life. Further treatment considerations, such as medication, surgery, pain management, and drug trials, occur during the patient journey. Each treatment approach is different depending on the severity of disease and the primary symptomatic manifestation affecting the patient, and the overall treatment plan will be based on the experience of the multidisciplinary team. In the authors’ experience, less-invasive options, such as sclerotherapy, embolization, laser ablation, and medical management (including systemic therapies such as sirolimus), have begun to replace surgical procedures if the multidisciplinary team feels the medical needs of the patient can be met. When patients begin puberty, additional treatment considerations may be needed for genetic testing and contraceptives; some patients with PROS conditions are at risk of venous thromboembolism; therefore, combined oral contraception would be contraindicated [9, 19]. During adulthood, as patients gain control of their care, they may begin doing further research into treatment options, particularly those that have become available since their childhood. At this stage, some patients may have concerns that medication or surgery could worsen their condition. A panel discussion in which the authors discuss transitioning to adult care is provided in Additional file 4.

### Care team

Patients treated in community hospitals are more likely to have multiple specialties managing their care than patients in academic hospitals. Among patients whose treatment was led by a single practitioner, orthopedic surgery and genetics were the most common specialties identified. Among patients with a multidisciplinary care team, such as those being treated at a vascular anomaly center, the most common leading specialties were pediatrics, hematology/oncology, and general surgery. Because of the early onset of PROS, some patients may be treated long-term by a pediatric team, but eventually need to find specialists with experience treating adult patients. On the occasions when patients with PROS conditions need to interact with HCPs outside of their own care team (eg, dentists, urgent care, emergency room), it can be especially challenging for patients and families to convey the details and seriousness of their medical condition. A panel discussion in which the authors discuss finding the right care team is provided in Additional file 5.

### Financial pressures

Paying medical bills is a constant pressure, along with repeated follow-up with medical offices and insurance companies. Patients and families must pay close attention to their health insurance plans to ensure the necessary specialists are included. Patients often need to travel for specialist care, incurring costs for travel, accommodation, and meals. Reduction in employment is an additional financial complication; often one parent must quit their job to become a full-time caregiver. Additional financial challenges are described in Tables 2 and 3.

### Social challenges, emotional support, and mental health

Parents often feel alone after their child has been diagnosed with a PROS condition and experience a range of emotional and psychosocial challenges (Table 4). Families may be restricted in their ability to socialize and engage in common activities because of their child’s medical condition and physical limitations. Finding an established advocacy group permits parents to connect with others for resources, information, support, and advice. Meeting another patient with the same condition is an important experience, but such meetings can be difficult to arrange due to the rarity of PROS conditions and the expense of travel. The patient’s mental health may not be adequately supported by the primary care team that is concerned with treating the physical manifestations of the disease. Depression and anxiety are common, especially as patients get older. Risk of malignancy, although occurrences are rare [20], may contribute to ongoing anxiety and reduced quality of life. A panel discussion in which the authors discuss mental health and quality of life is provided in Additional file 6.

**Table 4:** Emotional and psychosocial challenges during the patient journey

Stage of life	Emotional/psychosocial challenges across PROS
Birth to toddler	Particularly challenging period for caregivers Searching for answers is overwhelming and stressful Many struggle with discussing with friends and family Concerns about having other children Frustration with lack of treatment options Constant worry and uncertainty
Elementary school	Patients face constant staring, questions, assumptions of disability Patients may experience frustration with limitations on activities and clothes/shoes For more severe cases, caregivers may need to cope with realization that patient’s condition will not improve significantly
Puberty/teenager	Patients struggle with peer acceptance Some patients struggle with mental health and anxiety (despite strong support systems) Caregivers are concerned about patient well-being at this difficult stage of life Patients may experience anger and self-consciousness; less willing to discuss condition For some patients, difficulty finding trendy clothing due to larger extremities Caregivers have concern and uncertainty about behavior issues (when applicable)
Adulthood	Patients have challenges finding new doctors and constantly explaining condition For patients, the planning needed to manage care can be exhausting Patients and caregivers are excited about independence but concerned about health issues Stress for caregiver if patient requires constant care into adulthood (contingency planning, etc.) Depending on severity and challenges they face, independent patients without strong support systems may struggle

PROS, *PIK3CA*-related overgrowth spectrum

### The PROS community

Shared support is very important in the rare disease setting. Advocacy groups provide an essential service, allowing families to connect with and learn from each other and share their experiences, most commonly through online forums. Despite a shared genetic mutation across the PROS conditions, the variety of ways in which the phenotypes present makes it challenging to create a wider community of patients with PROS. Many patients share the commonality of lifelong medical intervention, physical differences, disability, and pain*;* however, patients identify more with their individual phenotype, and many PROS conditions do not manifest similar symptoms, leading to lack of shared experiences. Advocacy groups are organized by PROS condition (eg, CLOVES Syndrome Community or K-T Support Group), making it challenging for patients with different conditions to connect. Even within the same disorder, patients may experience mild or very severe manifestations. Patients with a single isolated lesion may not relate well to patients with extensive disease. Multiple names for the same condition (eg, M-CM and MCAP; FH and FAO) may make it difficult for patients to connect with each other. The term “PROS” as a diagnosis can make it challenging for patients to find the correct advocacy group for their specific issues and symptoms. The lack of a central location for PROS information and resources is a significant challenge for HCPs, patients, and families.

## Discussion

The aim of this research is to increase awareness of PROS and educate on the complex issues faced by patients and families, and in doing so, improve the experiences and health care of patients. The PROS patient experience journey was developed with close collaboration among patients, caregivers, and patient advocates. By mapping the journey from the viewpoint of the patient rather than the health care system, this novel methodology, which could be applied elsewhere, can accurately identify areas of unmet need, barriers to care, education topics, and also assist HCPs in understanding the patient and family perspective.

Our approach had certain limitations. To our knowledge this is the first time a project like this has been attempted to define the patient and caregiver experience in PROS. Our HCP research was limited to physicians in the US, Germany, and France, and is therefore reflective of clinical practice in those countries only. The ethnographic research was US-based and focused on patients with K–T syndrome, CLOVES syndrome, and M-CM, and therefore did not fully capture patient experiences outside the US or from the full spectrum of PROS disorders. Nonetheless, we feel that we have obtained a unique insight into the patient and caregiver experience in PROS and have identified several opportunities to support the PROS community.

Patient and caregiver support could be enhanced by development of patient-friendly, accurate educational materials. These materials could be available for parents to provide to schools, non-expert HCPs, and allied health providers to help families communicate with others about PROS conditions. Infographic or other visual resources would be helpful to explain challenging topics, such as how the *PIK3CA* mutation occurs and how genetic testing is performed. Improved access to resources and specialists for patients and family emotional health needs would be welcomed, as would opportunities for patients to meet each other. Access to support for the transition from pediatric to adult care is a key unmet need due to an insufficient number of HCPs with training and experience in how to treat adult patients with PROS conditions. Opportunities to share real-world patient information include patient registries, webinars, and regional multidisciplinary conferences organized by vascular anomaly centers, children’s hospitals, and patient advocacy groups. Proceedings of regional conferences could be published in multidisciplinary journals in patient-accessible language.

Lack of evidence in the literature to support treatment algorithms and best options for initial therapy is a major unmet need. Opportunities for HCP support therefore include updated diagnosis guidelines, updated treatment guidelines based on multidisciplinary experience, improved pain management guidelines, real-world outcomes research, and tools for HCPs to easily refer patients to advocacy groups. Opportunities for health care systems include improved care coordination and case management, and improved access to remote services, such as telemedicine. Opportunities for support of advocacy groups include a database of PROS specialists and treatment centers and high-quality educational tools to share with patients. Patients and advocates should be equal partners in creating and validating patient journeys in PROS and other rare, challenging conditions.

## Supplementary Information

**Additional file 1**. Patient journey.

**Additional file 2.**. Path to diagnosis.

**Additional file 3**. Treatment decision making.

**Additional file 4**. Transitioning to adult care.

**Additional file 5**. Finding the right care team.

**Additional file 6**. Mental Health and Quality of Life.

## Data Availability

The datasets used and/or analyzed during the current study are available from the corresponding author on reasonable request.

## Abbreviations

ABA: Applied behavioral analysis
AKT: Protein kinase B
CLA: Complex lymphatic anomaly
CLOVES: Congenital lipomatous overgrowth, vascular malformations, epidermal nevi, scoliosis/skeletal and spinal anomalies
DMEG: Dysplastic megalencephaly
DVT: Deep venous thrombosis
FAVA: Fibroadipose vascular anomaly
FH/FAO: Fibroadipose hyperplasia
FIL: Fibroadipose or facial infiltrating lipomatosis
FT: Feeding therapy
HCP: Health care professional
ILM: Isolated lymphatic malformation
ISSVA: International Society for the Study of Vascular Anomalies
K–T: Klippel–Trénaunay
M-CM/MCAP: Megalencephaly-capillary malformation syndrome
N/A: Not applicable
NIH: National Institutes of Health
NORD: National Organization for Rare Disorders
OT: Occupational therapy
PE: Pulmonary embolism
PI3K: Phosphatidylinositol-3-kinase
*PIK3CA*: Phosphatidylinositol-4,5-bisphosphate 3-kinase catalytic subunit alpha
PROS: *PIK3CA*-Related overgrowth spectrum
PT: Physical therapy
ST: Speech therapy
US: United States
