# Supplementary material for: Mapping the PIK3CA-related overgrowth spectrum (PROS) patient and caregiver journey using a patient-centered approach
Source: Orphanet J Rare Dis. 2022 May 7;17:189. doi: 10.1186/s13023-022-02338-1 (PMC9077929; doi:10.1186/s13023-022-02338-1)
Supplement: Supplementary file 1 — Additional file 1. Patient journey. [file 13023_2022_2338_MOESM1_ESM.pdf]

Everyone with a PROS condition is unique, but there are many common experiences.

Which patient journey would you like to explore?

THE PROS Journey

THE K-T Journey

THE CLOVES Journey

THE M-CM Journey

Everyone with a PROS condition is unique, but there are many common experiences.

Which patient journey would you like to explore?

THE PROS Journey

PROS (or *PIK3CA*-related overgrowth spectrum) is a wide-ranging spectrum of disorders caused by mutations in a gene called *PIK3CA*.

THE K-T Journey

THE CLOVES Journey

THE M-CM Journey

Choose where to begin the PROS journey ...

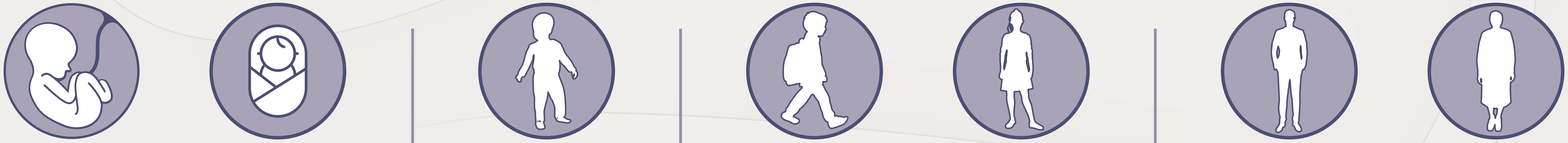

In Utero | Birth | Toddler/Preschool | Elementary School | Puberty/Teenager | Young Adulthood | Older Adulthood

THE PROS Journey

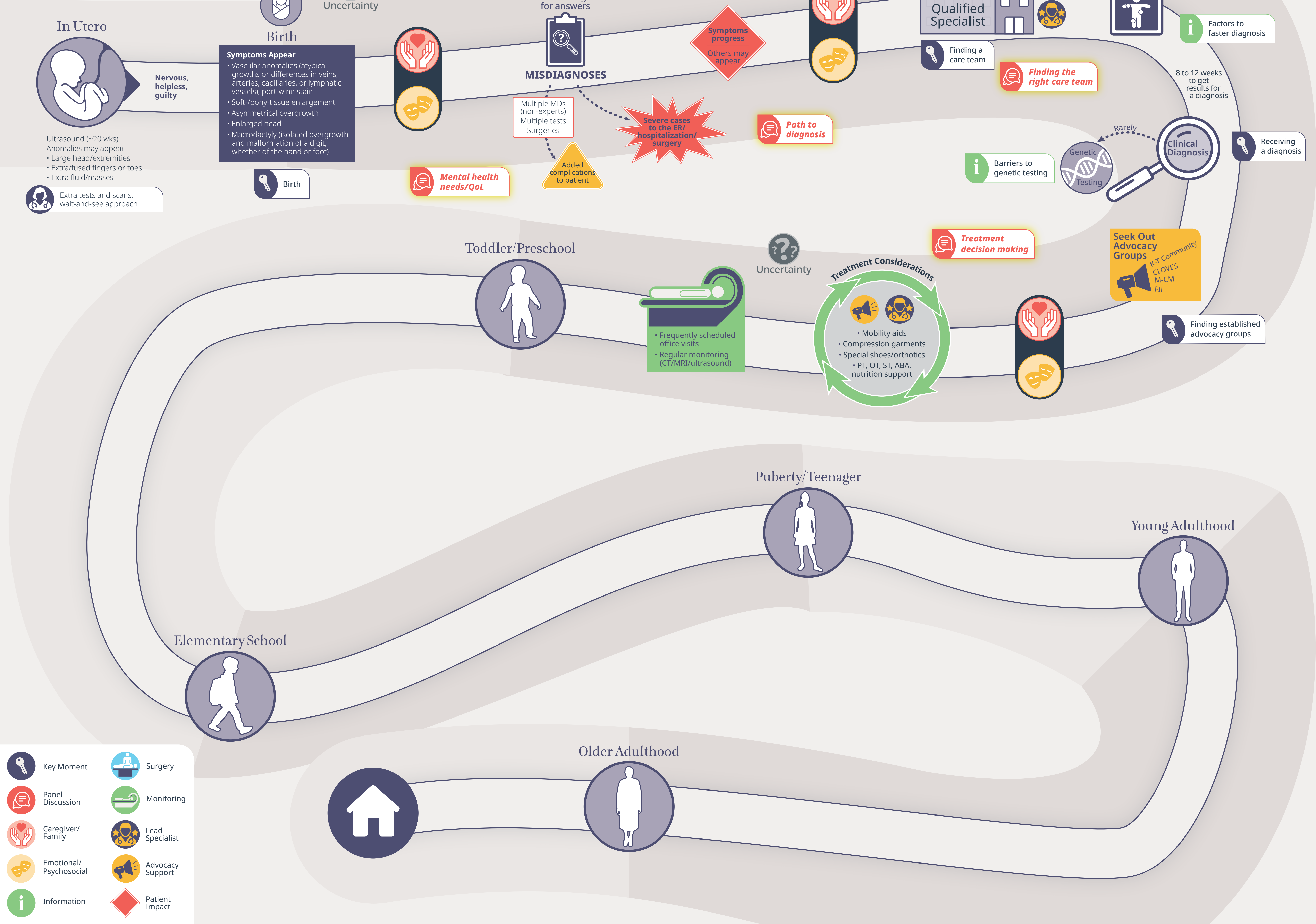

THE PROS Journey

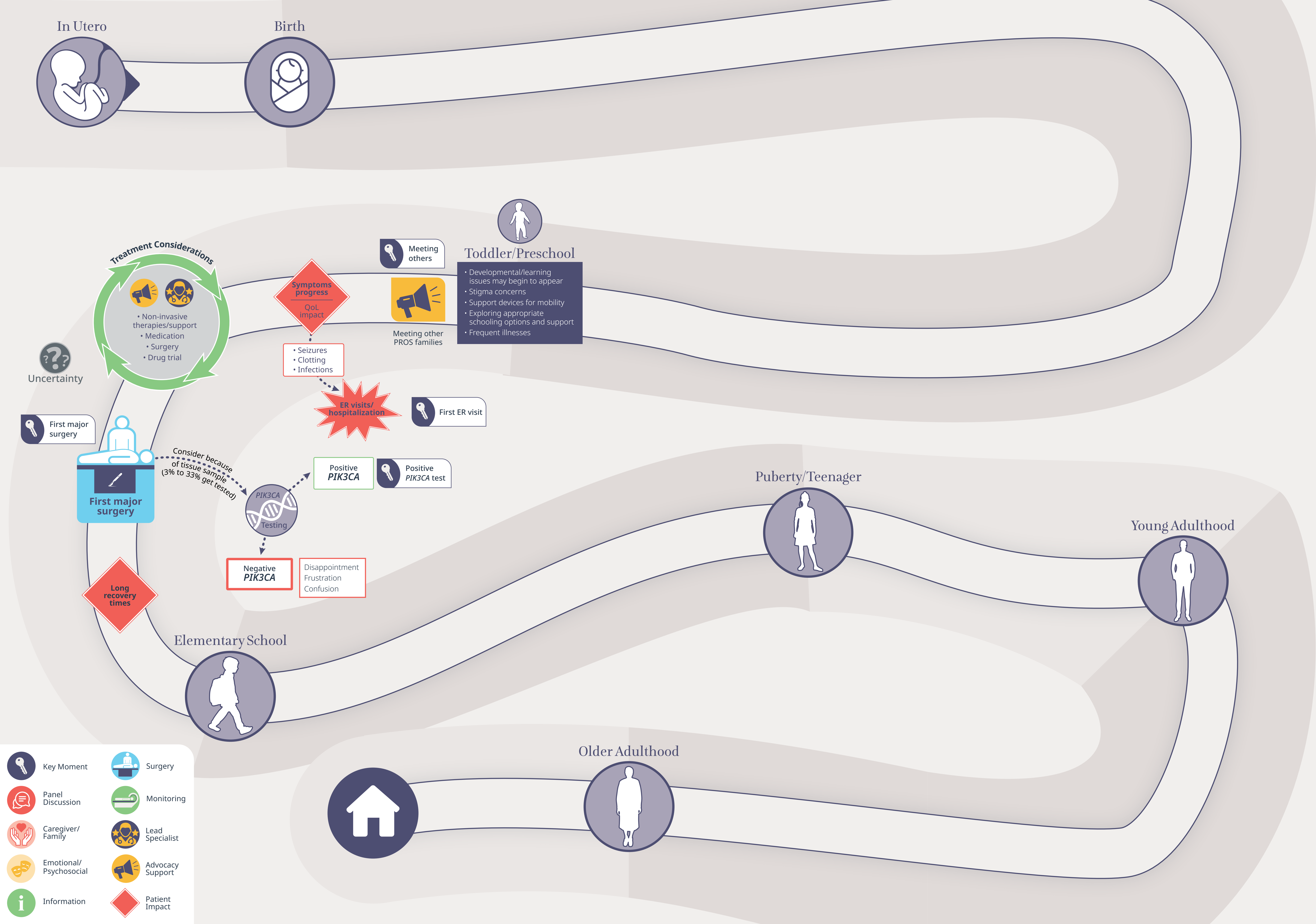

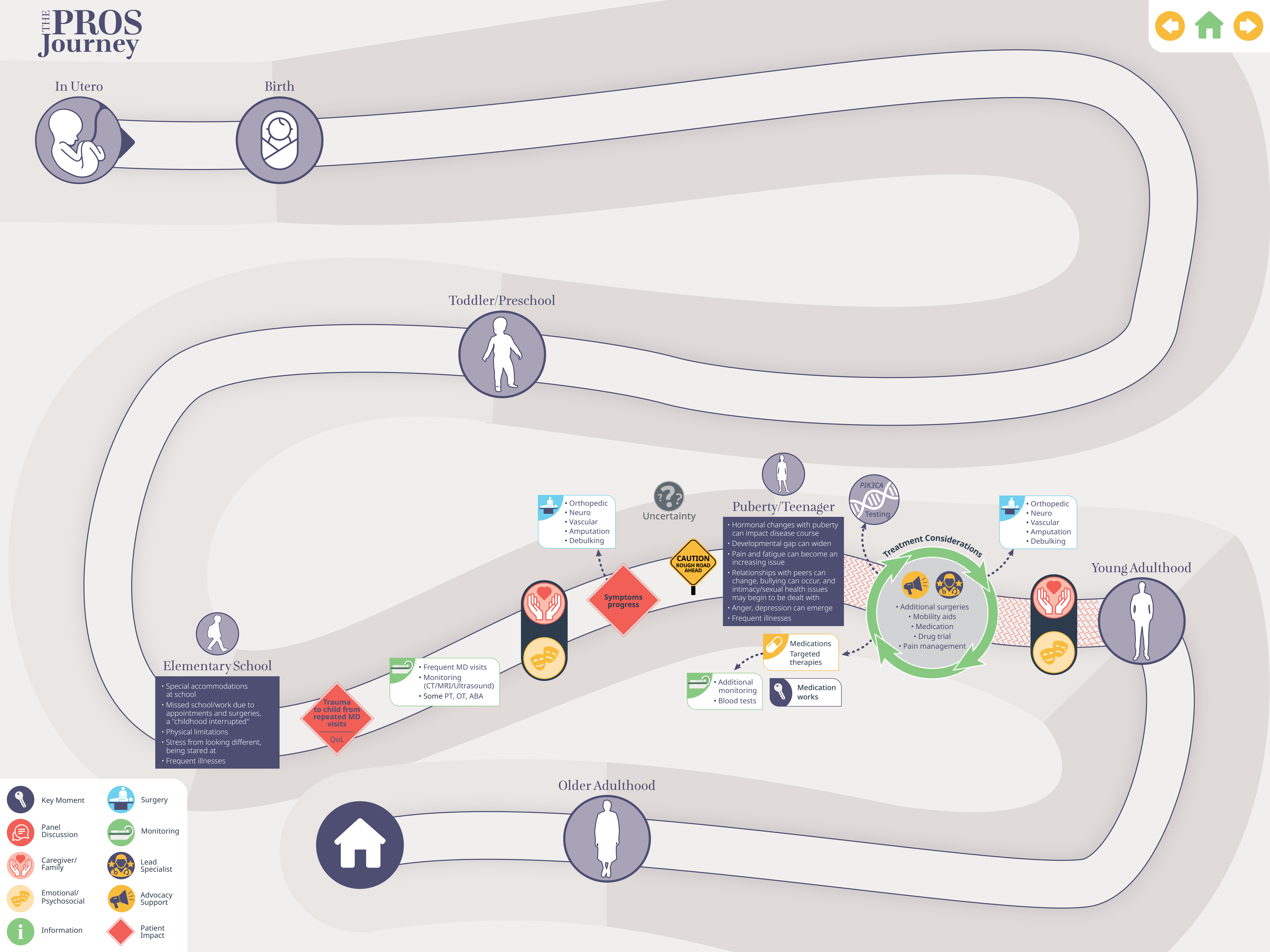
THE PROS Journey
In Utero
Birth
Toddler/Preschool
Elementary School
• Special accommodations at school
• Missed school/work due to appointments and surgeries, a "childhood interrupted"
• Physical limitations
• Stress from looking different, being stared at
• Frequent illnesses
Trauma to child from repeated MD visits
QoL
• Frequent MD visits
• Monitoring (CT/MRI/Ultrasound)
• Some PT, OT, ABA
• Orthopedic
• Neuro
• Vascular
• Amputation
• Debulking
Symptoms progress
Uncertainty
CAUTION ROUGH ROAD AHEAD
Puberty/Teenager
• Hormonal changes with puberty can impact disease course
• Developmental gap can widen
• Pain and fatigue can become an increasing issue
• Relationships with peers can change, bullying can occur, and intimacy/sexual health issues may begin to be dealt with
• Anger, depression can emerge
• Frequent illnesses
PIK3CA
Testing
Treatment Considerations
• Additional surgeries
• Mobility aids
• Medication
• Drug trial
• Pain management
Medications
Targeted therapies
• Additional monitoring
• Blood tests
Medication works
• Orthopedic
• Neuro
• Vascular
• Amputation
• Debulking
Young Adulthood
Older Adulthood
Key Moment
Panel Discussion
Caregiver/ Family
Emotional/ Psychosocial
Information
Surgery
Monitoring
Lead Specialist
Advocacy Support
Patient Impact

THE PROS Journey

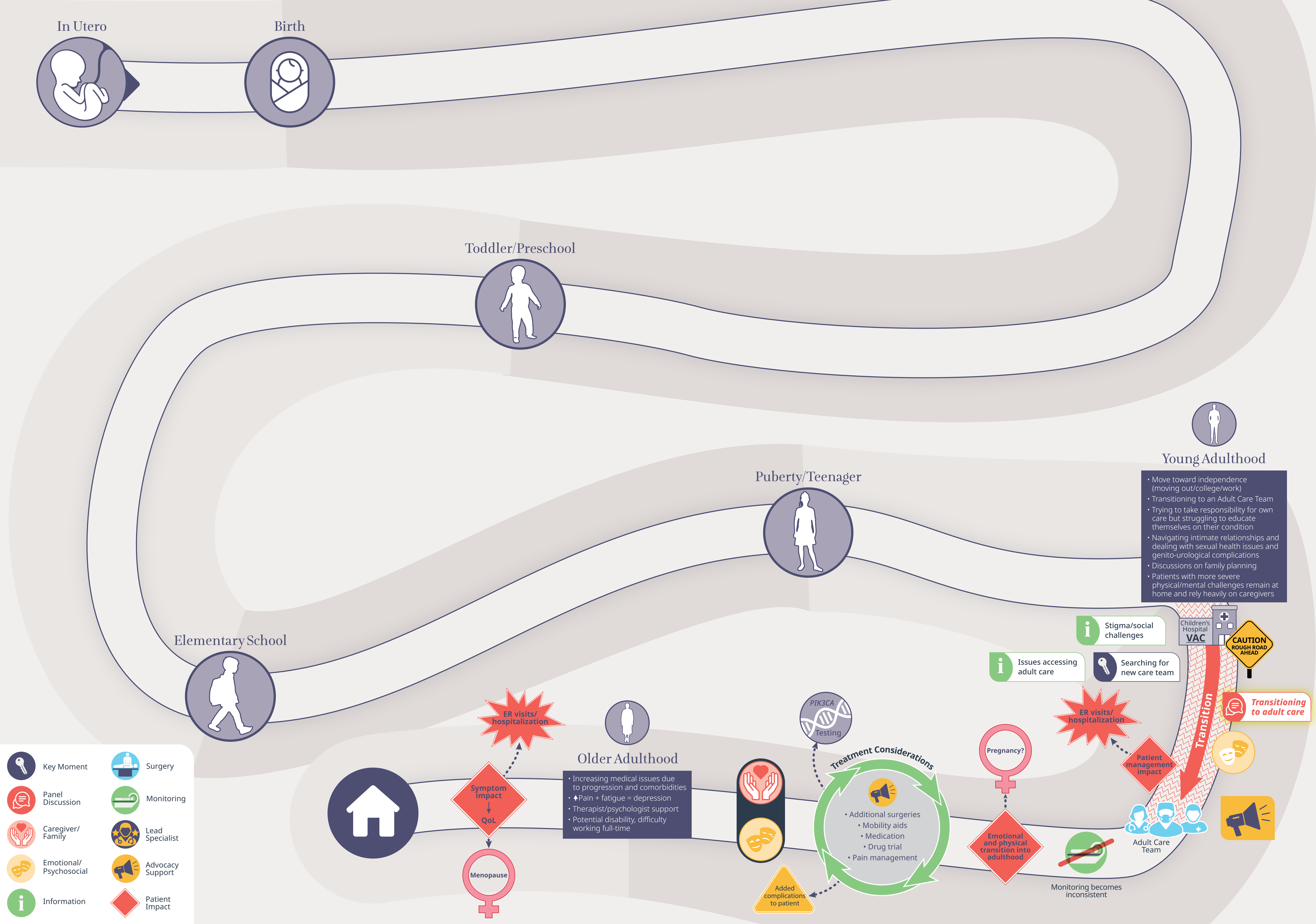

Everyone with a PROS condition is unique, but there are many common experiences.

Which patient journey would you like to explore?

THE PROS Journey

THE K-T Journey

THE CLOVES Journey

THE M-CM Journey

Klippel-Trenaunay (K-T) syndrome is characterized by a triad of capillary, lymphatic, and venous malformations. In most cases overgrowth is present.

Choose where to begin the K-T journey ...

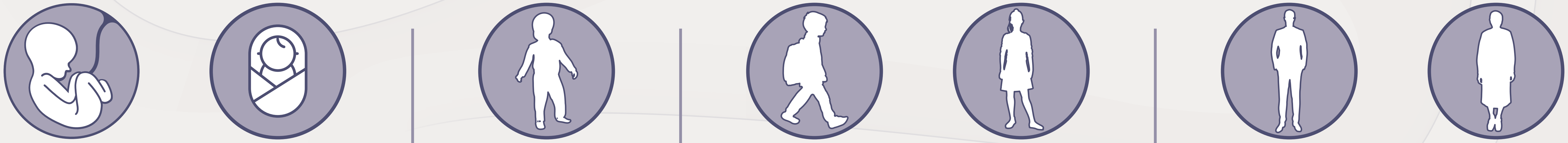

In Utero | Birth | Toddler/Preschool | Elementary School | Puberty/Teenager | Young Adulthood | Older Adulthood

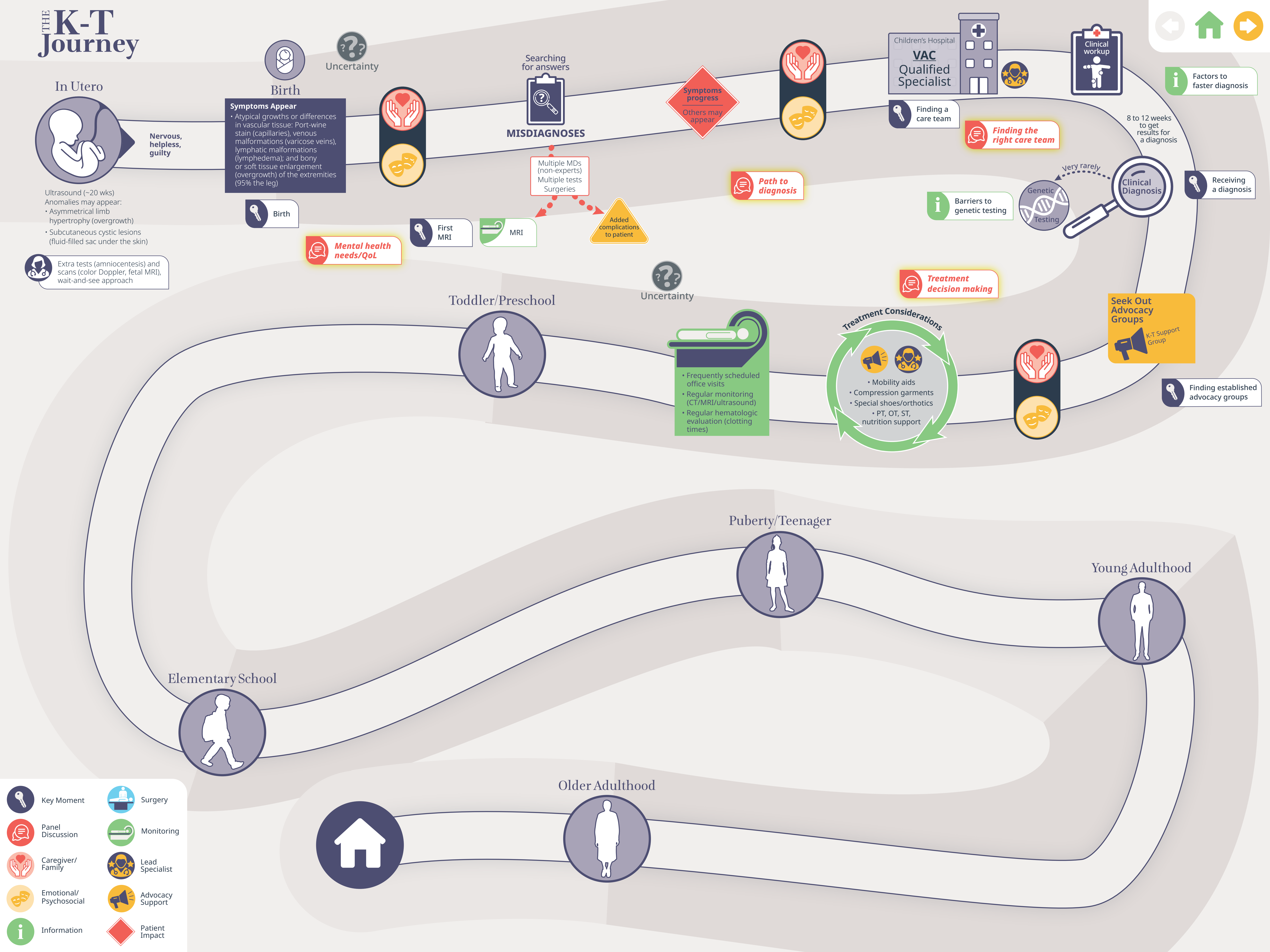

THE K-T Journey
In Utero
Nervous, helpless, guilty
Ultrasound (~20 wks)
Anomalies may appear:
• Asymmetrical limb hypertrophy (overgrowth)
• Subcutaneous cystic lesions (fluid-filled sac under the skin)
Extra tests (amniocentesis) and scans (color Doppler, fetal MRI), wait-and-see approach
Birth
Uncertainty
Symptoms Appear
• Atypical growths or differences in vascular tissue: Port-wine stain (capillaries), venous malformations (varicose veins), lymphatic malformations (lymphedema); and bony or soft tissue enlargement (overgrowth) of the extremities (95% the leg)
Birth
Mental health needs/QoL
First MRI
MRI
Searching for answers
MISDIAGNOSES
Multiple MDs (non-experts)
Multiple tests
Surgeries
Added complications to patient
Symptoms progress
Others may appear
Path to diagnosis
Children's Hospital
VAC
Qualified Specialist
Finding a care team
Finding the right care team
Clinical workup
Factors to faster diagnosis
8 to 12 weeks to get results for a diagnosis
Very rarely
Clinical Diagnosis
Genetic Testing
Barriers to genetic testing
Receiving a diagnosis
Uncertainty
Treatment decision making
Seek Out Advocacy Groups
K-T Support Group
Finding established advocacy groups
Toddler/Preschool
• Frequently scheduled office visits
• Regular monitoring (CT/MRI/ultrasound)
• Regular hematologic evaluation (clotting times)
Treatment Considerations
• Mobility aids
• Compression garments
• Special shoes/orthotics
• PT, OT, ST, nutrition support
Puberty/Teenager
Young Adulthood
Elementary School
Older Adulthood
Key Moment
Surgery
Panel Discussion
Monitoring
Caregiver/ Family
Lead Specialist
Emotional/ Psychosocial
Advocacy Support
Information
Patient Impact

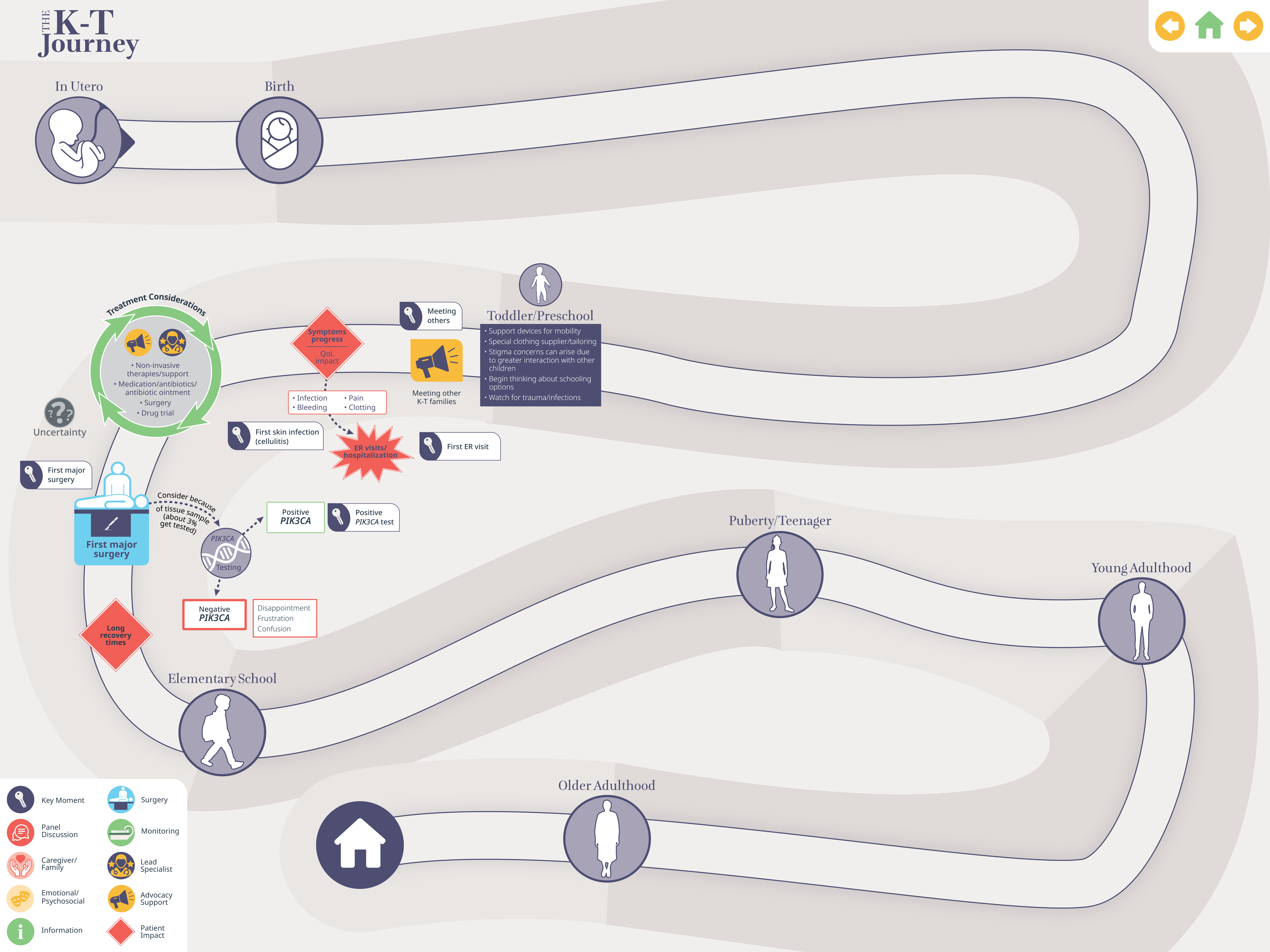

THE K-T Journey
In Utero
Birth
Toddler/Preschool
• Support devices for mobility
• Special clothing supplier/tailoring
• Stigma concerns can arise due to greater interaction with other children
• Begin thinking about schooling options
• Watch for trauma/infections
Meeting others
Meeting other K-T families
Symptoms progress
QoL impact
• Infection
• Bleeding
• Pain
• Clotting
First skin infection (cellulitis)
ER visits/ hospitalization
First ER visit
Treatment Considerations
• Non-invasive therapies/support
• Medication/antibiotics/ antibiotic ointment
• Surgery
• Drug trial
Uncertainty
First major surgery
First major surgery
Consider because of tissue sample (about 3% get tested)
PIK3CA Testing
Positive PIK3CA
Positive PIK3CA test
Negative PIK3CA
Disappointment
Frustration
Confusion
Long recovery times
Elementary School
Puberty/Teenager
Young Adulthood
Older Adulthood
Key Moment
Surgery
Panel Discussion
Monitoring
Caregiver/ Family
Lead Specialist
Emotional/ Psychosocial
Advocacy Support
Information
Patient Impact

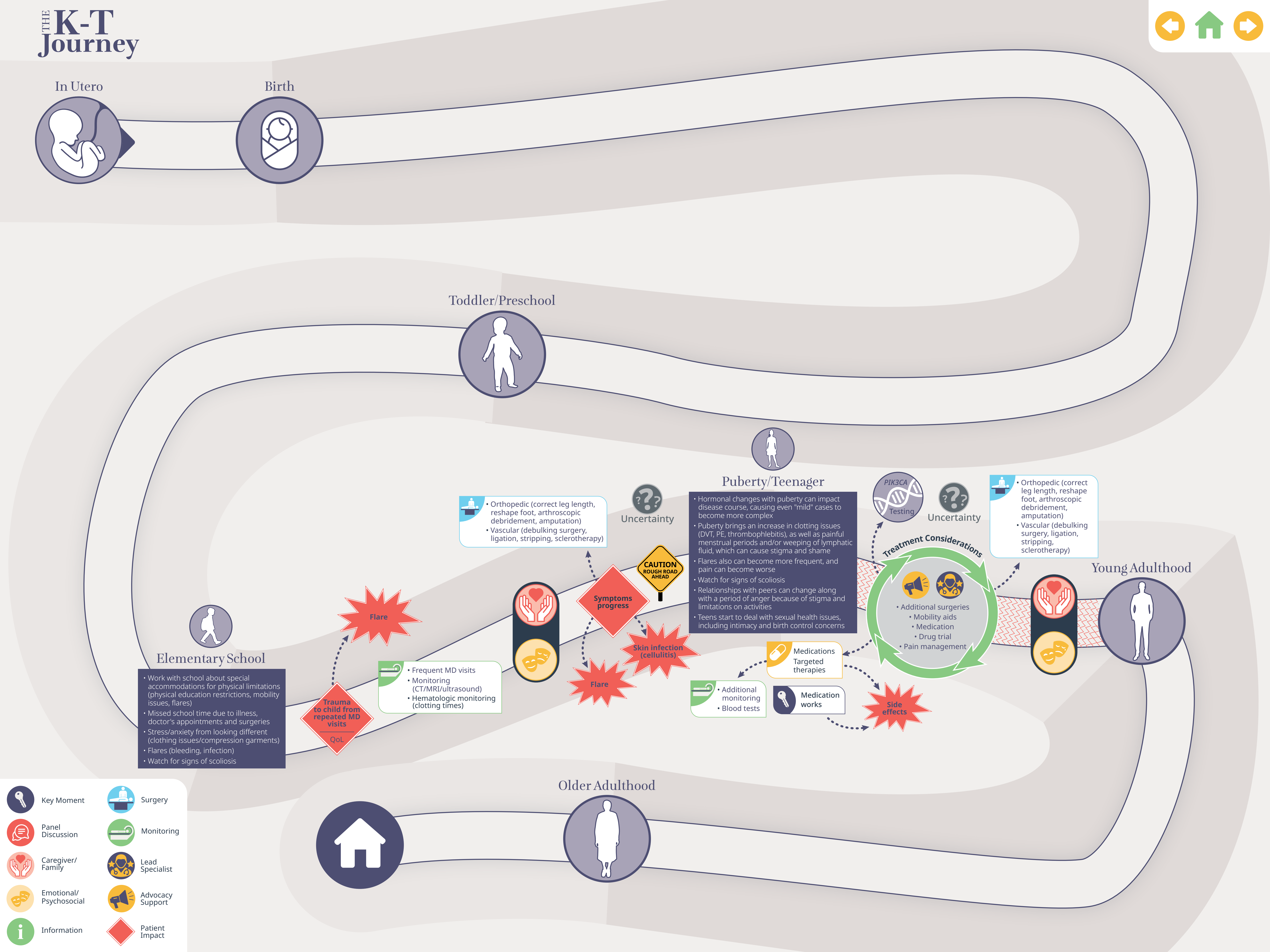
THE K-T Journey
In Utero
Birth
Toddler/Preschool
Elementary School
• Work with school about special accommodations for physical limitations (physical education restrictions, mobility issues, flares)
• Missed school time due to illness, doctor's appointments and surgeries
• Stress/anxiety from looking different (clothing issues/compression garments)
• Flares (bleeding, infection)
• Watch for signs of scoliosis
Trauma to child from repeated MD visits
QoL
Flare
• Frequent MD visits
• Monitoring (CT/MRI/ultrasound)
• Hematologic monitoring (clotting times)
• Orthopedic (correct leg length, reshape foot, arthroscopic debridement, amputation)
• Vascular (debulking surgery, ligation, stripping, sclerotherapy)
Uncertainty
Symptoms progress
CAUTION ROUGH ROAD AHEAD
Skin infection (cellulitis)
Flare
Puberty/Teenager
• Hormonal changes with puberty can impact disease course, causing even "mild" cases to become more complex
• Puberty brings an increase in clotting issues (DVT, PE, thrombophlebitis), as well as painful menstrual periods and/or weeping of lymphatic fluid, which can cause stigma and shame
• Flares also can become more frequent, and pain can become worse
• Watch for signs of scoliosis
• Relationships with peers can change along with a period of anger because of stigma and limitations on activities
• Teens start to deal with sexual health issues, including intimacy and birth control concerns
PIK3CA
Testing
Uncertainty
Treatment Considerations
• Additional surgeries
• Mobility aids
• Medication
• Drug trial
• Pain management
• Orthopedic (correct leg length, reshape foot, arthroscopic debridement, amputation)
• Vascular (debulking surgery, ligation, stripping, sclerotherapy)
Medications
Targeted therapies
• Additional monitoring
• Blood tests
Medication works
Side effects
Young Adulthood
Older Adulthood
Key Moment
Panel Discussion
Caregiver/ Family
Emotional/ Psychosocial
Information
Surgery
Monitoring
Lead Specialist
Advocacy Support
Patient Impact

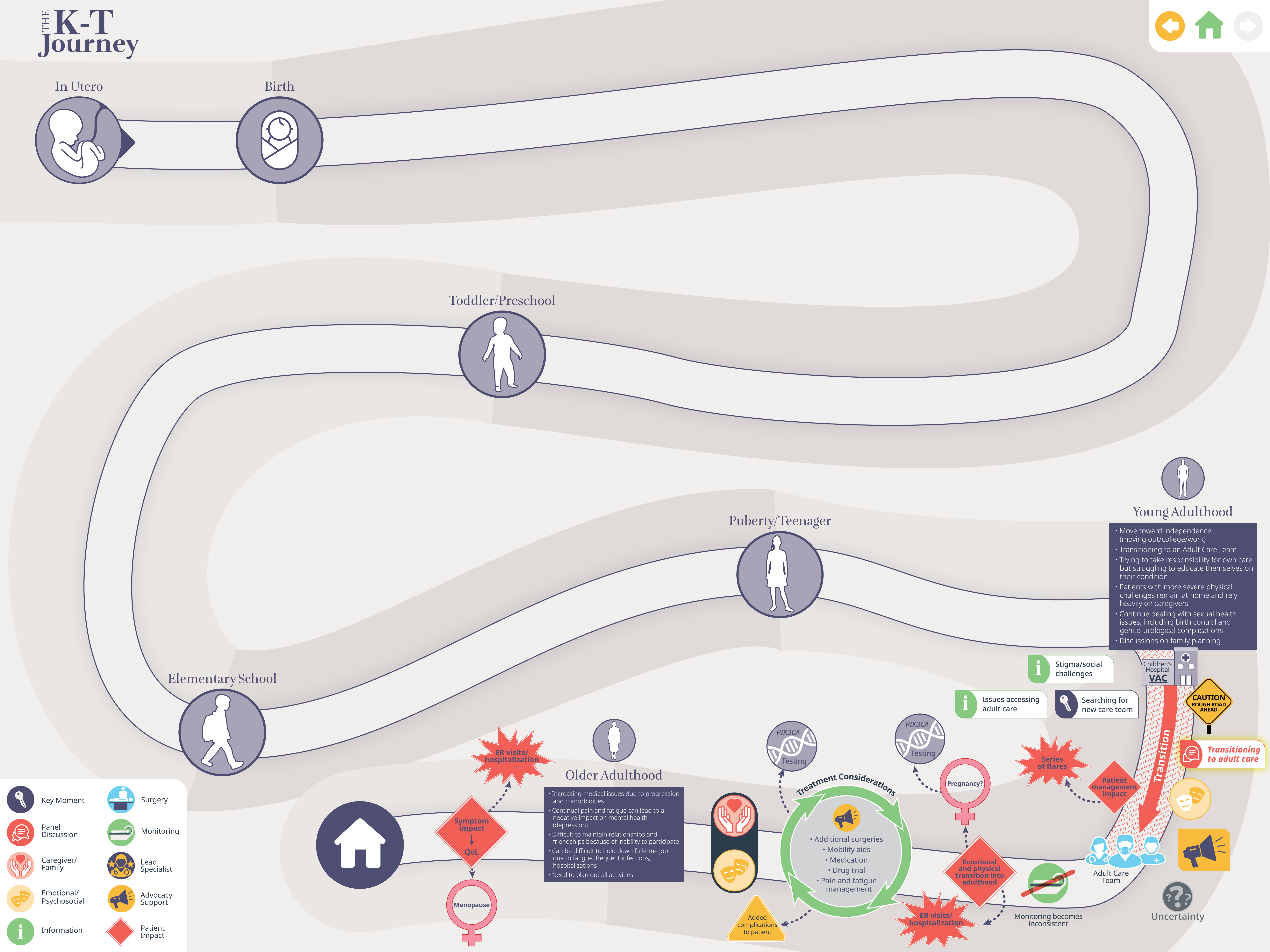

THE K-T Journey
In Utero
Birth
Toddler/Preschool
Puberty/Teenager
Elementary School
Young Adulthood
• Move toward independence (moving out/college/work)
• Transitioning to an Adult Care Team
• Trying to take responsibility for own care but struggling to educate themselves on their condition
• Patients with more severe physical challenges remain at home and rely heavily on caregivers
• Continue dealing with sexual health issues, including birth control and genito-urological complications
• Discussions on family planning
Stigma/social challenges
Children's Hospital
VAC
Issues accessing adult care
Searching for new care team
CAUTION ROUGH ROAD AHEAD
Transition
Transitioning to adult care
Series of flares
Patient management impact
Adult Care Team
Uncertainty
Monitoring becomes inconsistent
PIK3CA
Testing
PIK3CA
Testing
Pregnancy?
Emotional and physical transition into adulthood
ER visits/ hospitalization
Treatment Considerations
• Additional surgeries
• Mobility aids
• Medication
• Drug trial
• Pain and fatigue management
Added complications to patient
Older Adulthood
• Increasing medical issues due to progression and comorbidities
• Continual pain and fatigue can lead to a negative impact on mental health (depression)
• Difficult to maintain relationships and friendships because of inability to participate
• Can be difficult to hold down full-time job due to fatigue, frequent infections, hospitalizations
• Need to plan out all activities
ER visits/ hospitalization
Symptom impact
QoL
Menopause
Key Moment
Panel Discussion
Caregiver/ Family
Emotional/ Psychosocial
Information
Surgery
Monitoring
Lead Specialist
Advocacy Support
Patient Impact

Everyone with a PROS condition is unique, but there are many common experiences.

Which patient journey would you like to explore?

THE PROS Journey

THE K-T Journey

THE CLOVES Journey

THE M-CM Journey

CLOVES syndrome is an overgrowth syndrome with complex vascular malformations. CLOVES stands for congenital, lipomatous overgrowth, vascular malformations, epidermal nevi and scoliosis/skeletal/spinal anomalies.

Choose where to begin the CLOVES journey ...

In Utero

Birth

Toddler/Preschool

Elementary School

Puberty/Teenager

Young Adulthood

Older Adulthood

THE CLOVES Journey

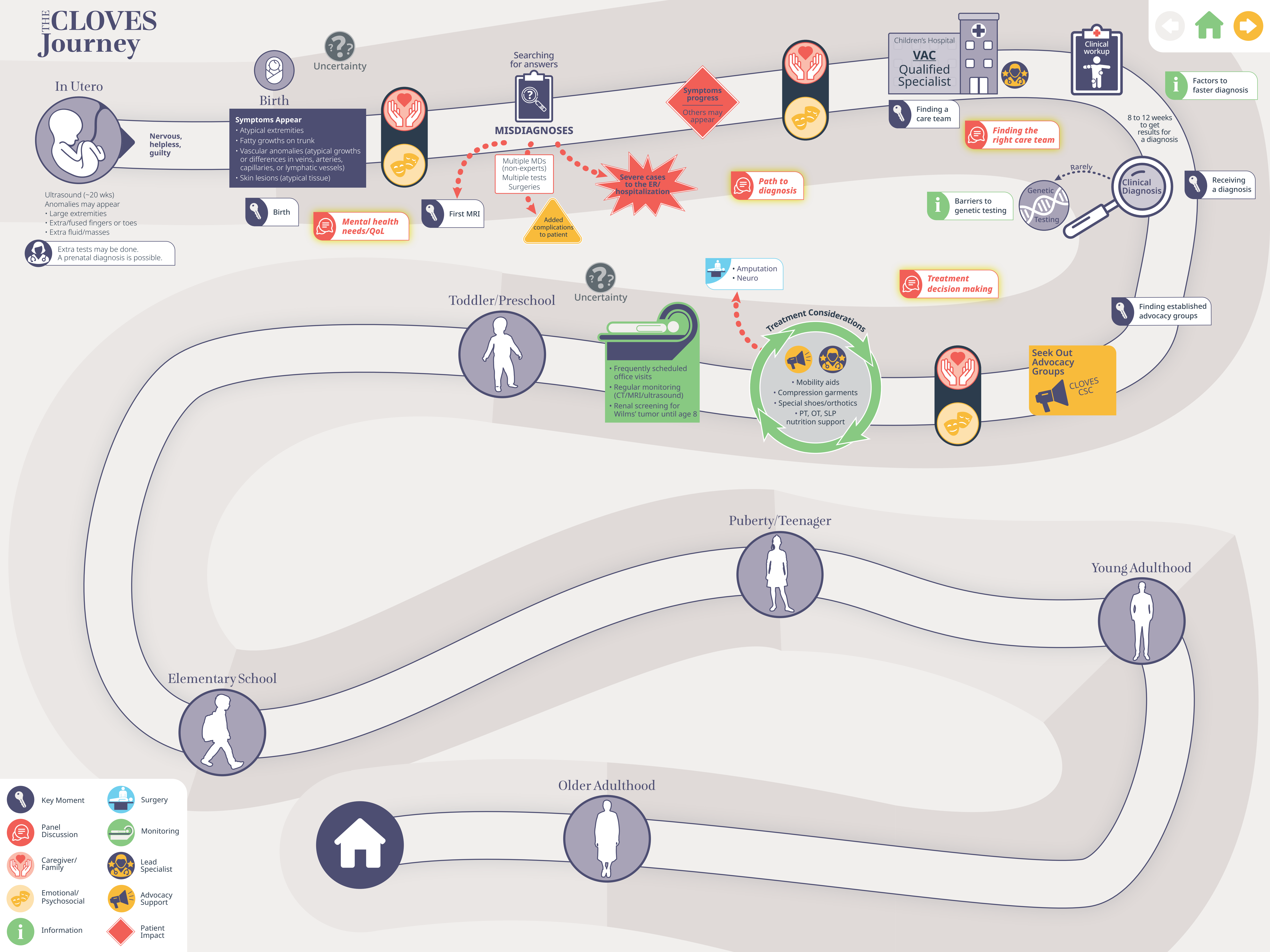

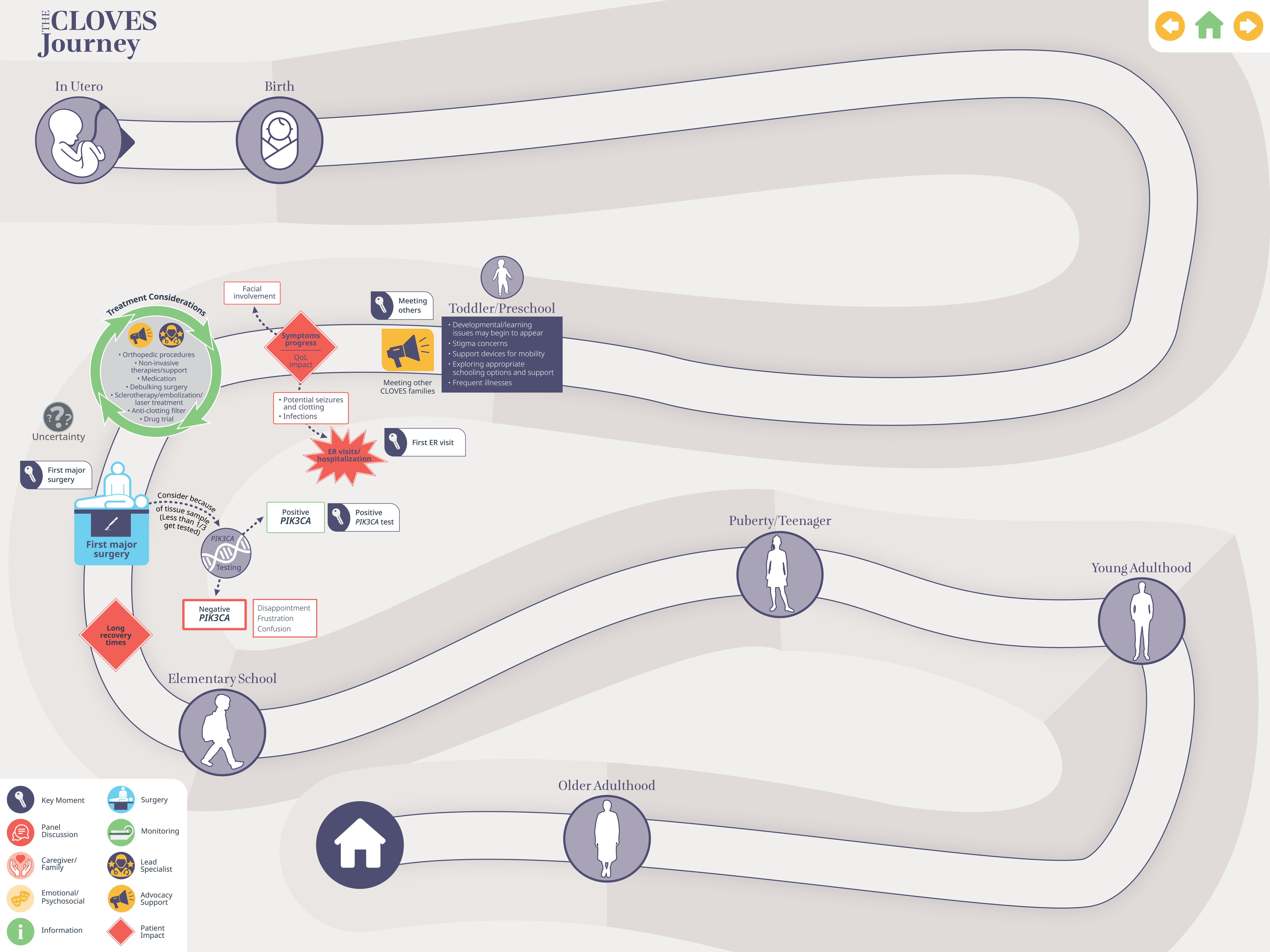
THE CLOVES Journey
In Utero
Birth
Toddler/Preschool
• Developmental/learning issues may begin to appear
• Stigma concerns
• Support devices for mobility
• Exploring appropriate schooling options and support
• Frequent illnesses
Meeting others
Meeting other CLOVES families
Facial involvement
Symptoms progress
QoL impact
• Potential seizures and clotting
• Infections
ER visits/ hospitalization
First ER visit
Treatment Considerations
• Orthopedic procedures
• Non-invasive therapies/support
• Medication
• Debulking surgery
• Sclerotherapy/embolization/ laser treatment
• Anti-clotting filter
• Drug trial
Uncertainty
First major surgery
First major surgery
Consider because of tissue sample (Less than 1/3 get tested)
PIK3CA Testing
Positive PIK3CA
Positive PIK3CA test
Negative PIK3CA
Disappointment
Frustration
Confusion
Long recovery times
Elementary School
Puberty/Teenager
Young Adulthood
Older Adulthood
Key Moment
Surgery
Panel Discussion
Monitoring
Caregiver/ Family
Lead Specialist
Emotional/ Psychosocial
Advocacy Support
Information
Patient Impact

THE CLOVES Journey

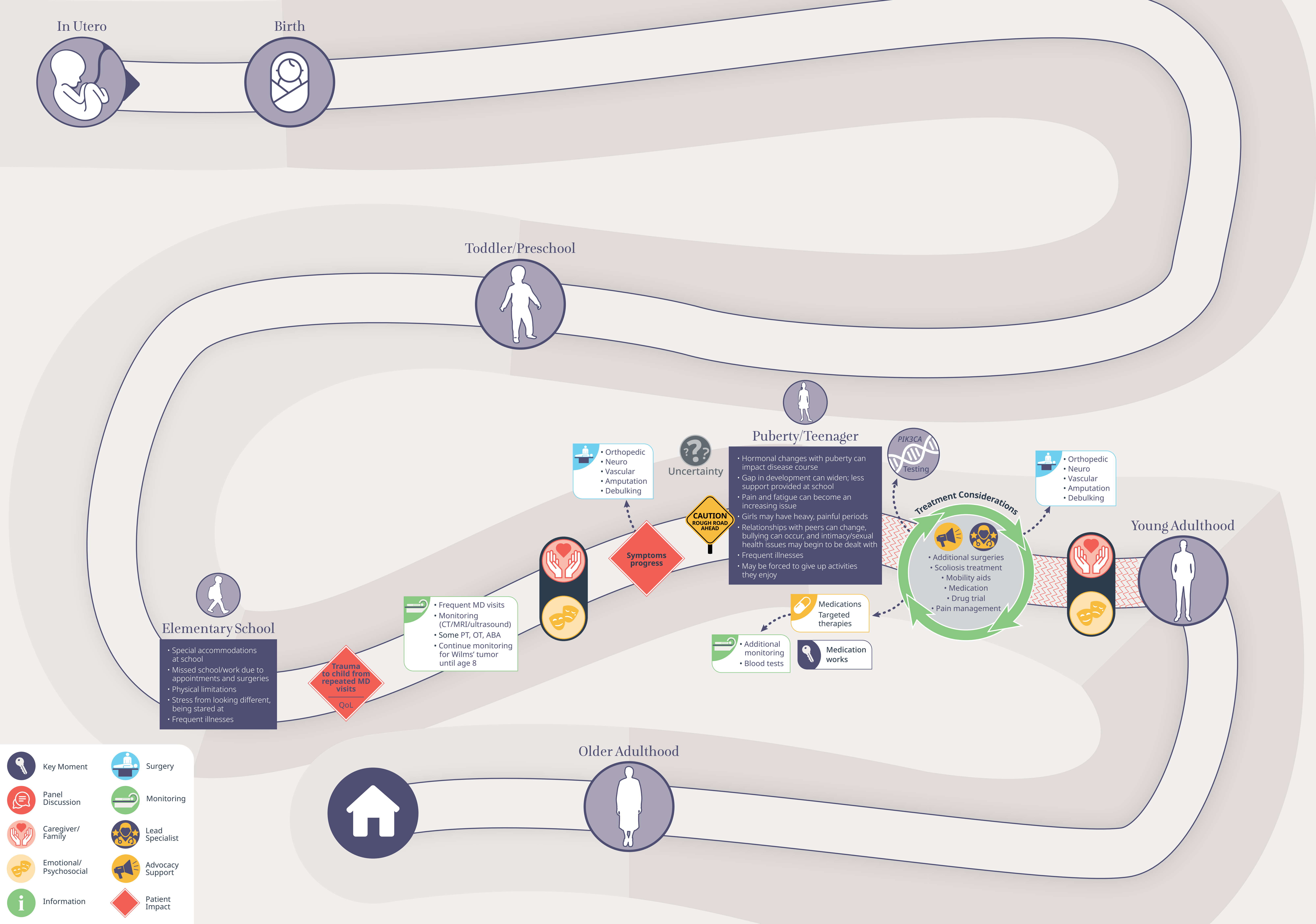

THE CLOVES Journey

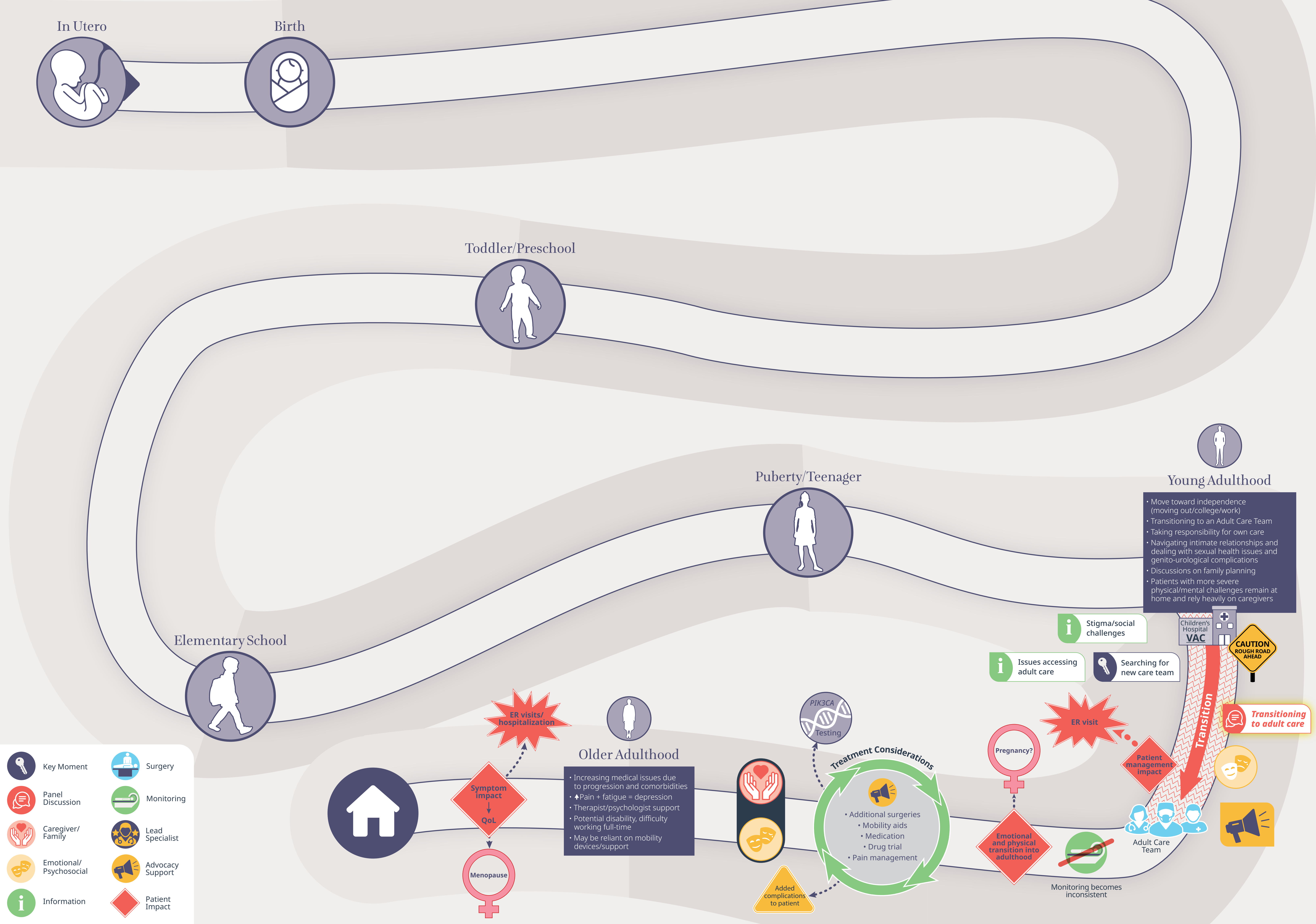

Everyone with a PROS condition is unique, but there are many common experiences.

Which patient journey would you like to explore?

THE PROS Journey

THE K-T Journey

THE CLOVES Journey

THE M-CM Journey

Macrocephaly-capillary malformation (M-CM) is a multiple malformation syndrome causing body and head overgrowth and irregularities of the skin, vascular system, brain, and limbs.

Choose where to begin the M-CM journey ...

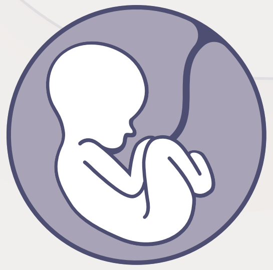

In Utero

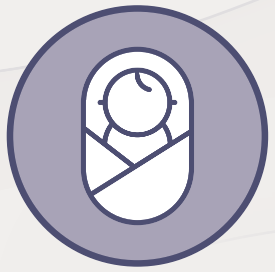

Birth

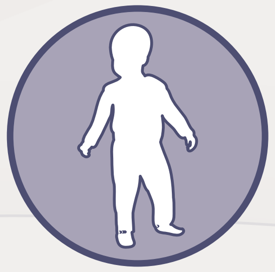

Toddler/Preschool

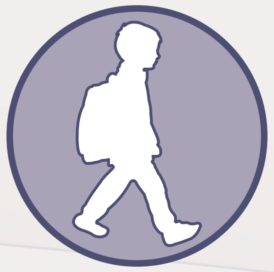

Elementary School

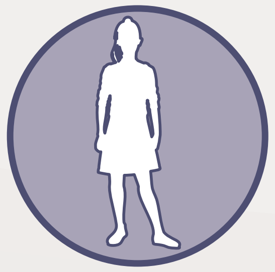

Puberty/Teenager

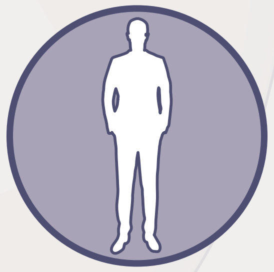

Young Adulthood

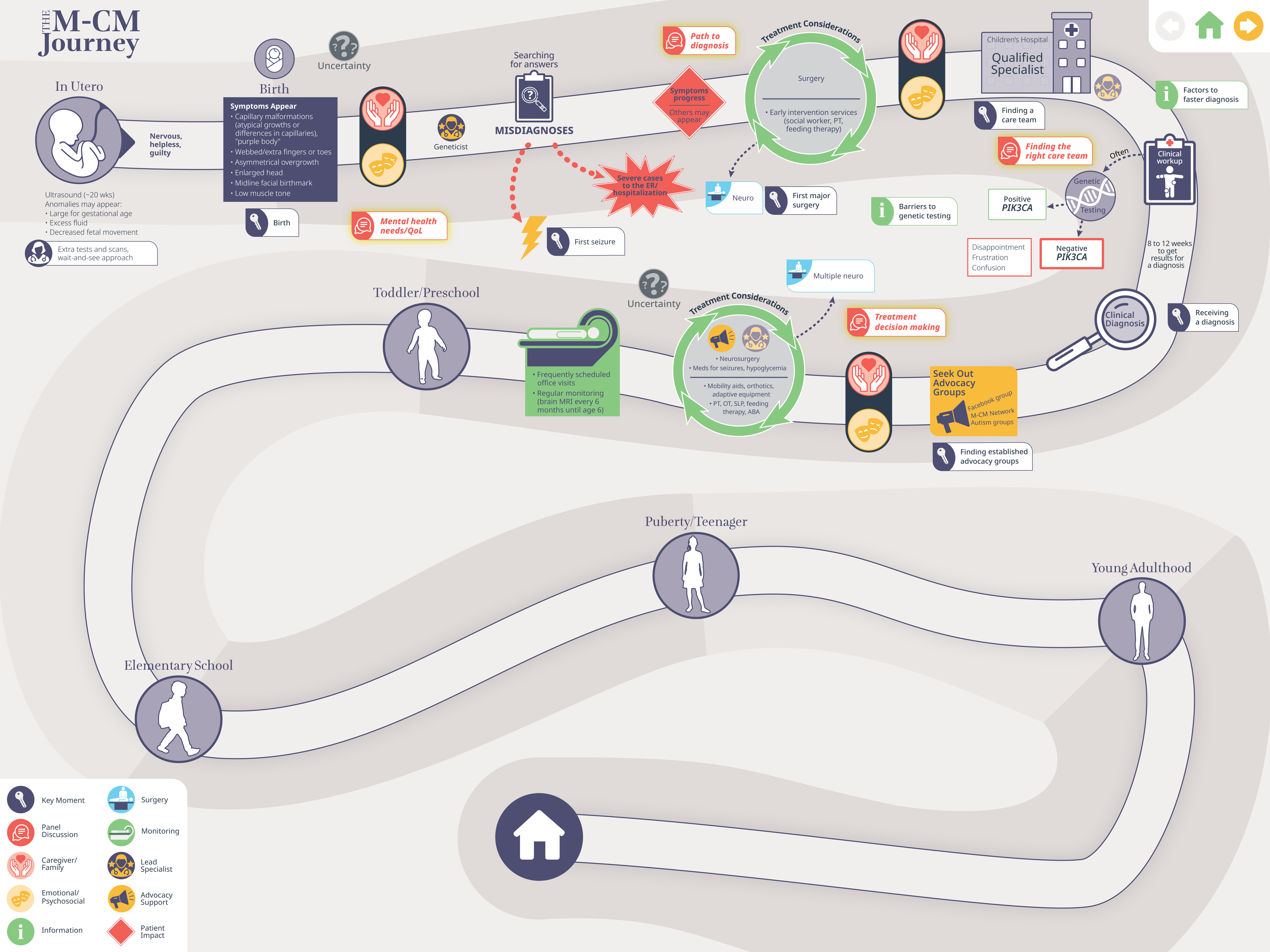
THE M-CM Journey
In Utero
Nervous, helpless, guilty
Ultrasound (~20 wks)
Anomalies may appear:
• Large for gestational age
• Excess fluid
• Decreased fetal movement
Extra tests and scans, wait-and-see approach
Birth
Symptoms Appear
• Capillary malformations (atypical growths or differences in capillaries), "purple body"
• Webbed/extra fingers or toes
• Asymmetrical overgrowth
• Enlarged head
• Midline facial birthmark
• Low muscle tone
Birth
Uncertainty
Mental health needs/QoL
Geneticist
Searching for answers
MISDIAGNOSES
Severe cases to the ER/ hospitalization
First seizure
Path to diagnosis
Symptoms progress
Others may appear
Treatment Considerations
Surgery
• Early intervention services (social worker, PT, feeding therapy)
Neuro
First major surgery
Barriers to genetic testing
Children's Hospital
Qualified Specialist
Finding a care team
Factors to faster diagnosis
Finding the right care team
Often
Clinical workup
Genetic Testing
Positive PIK3CA
Negative PIK3CA
Disappointment
Frustration
Confusion
8 to 12 weeks to get results for a diagnosis
Clinical Diagnosis
Receiving a diagnosis
Multiple neuro
Treatment decision making
Uncertainty
Treatment Considerations
• Neurosurgery
• Meds for seizures, hypoglycemia
• Mobility aids, orthotics, adaptive equipment
• PT, OT, SLP, feeding therapy, ABA
Toddler/Preschool
• Frequently scheduled office visits
• Regular monitoring (brain MRI every 6 months until age 6)
Seek Out Advocacy Groups
Facebook group
M-CM Network
Autism groups
Finding established advocacy groups
Puberty/Teenager
Young Adulthood
Elementary School
Key Moment
Surgery
Panel Discussion
Monitoring
Caregiver/ Family
Lead Specialist
Emotional/ Psychosocial
Advocacy Support
Information
Patient Impact

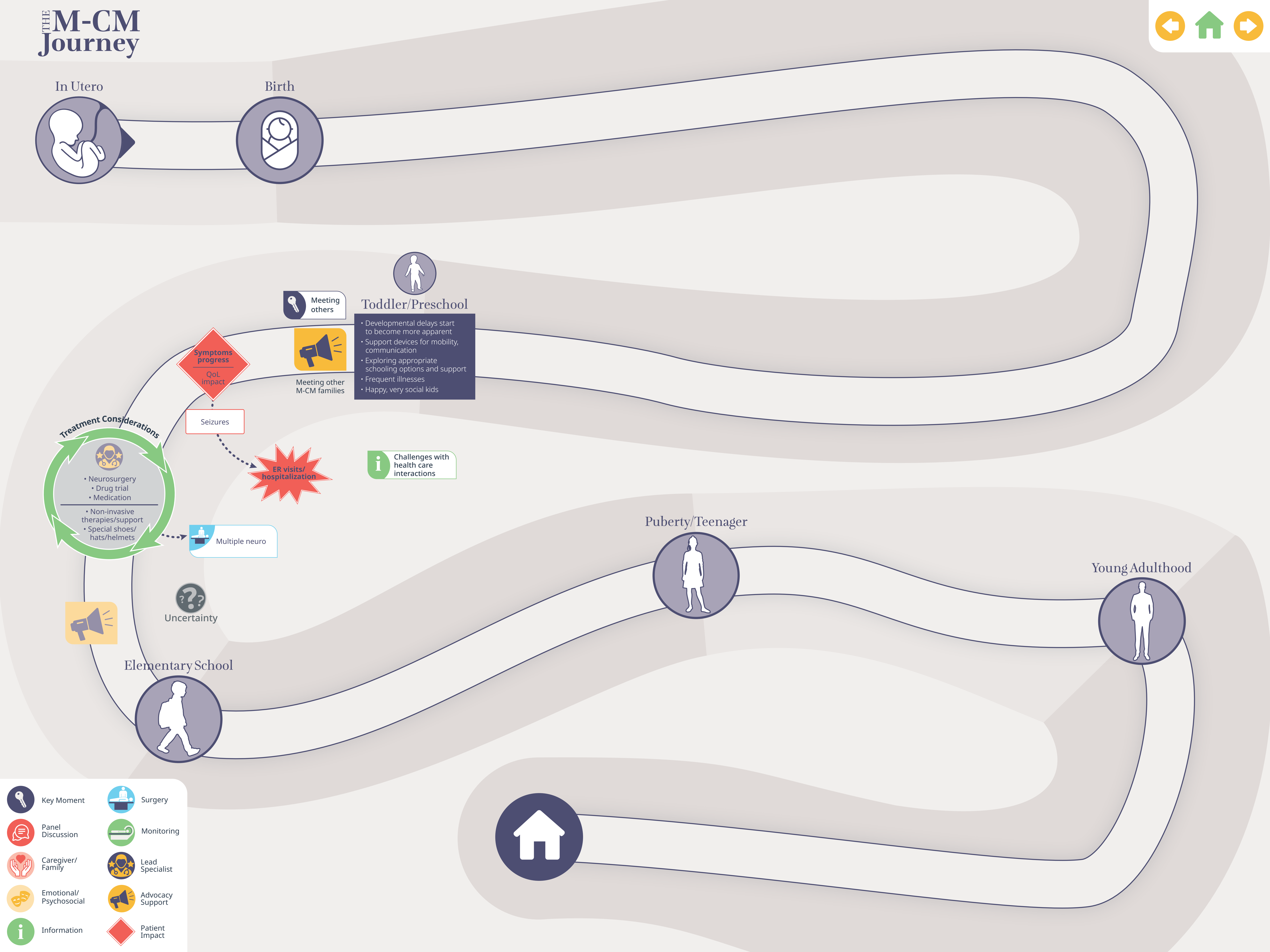

THE M-CM Journey
In Utero
Birth
Toddler/Preschool
• Developmental delays start to become more apparent
• Support devices for mobility, communication
• Exploring appropriate schooling options and support
• Frequent illnesses
• Happy, very social kids
Meeting others
Meeting other M-CM families
Symptoms progress
QoL impact
Seizures
ER visits/ hospitalization
Challenges with health care interactions
Treatment Considerations
• Neurosurgery
• Drug trial
• Medication
• Non-invasive therapies/support
• Special shoes/ hats/helmets
Multiple neuro
Uncertainty
Elementary School
Puberty/Teenager
Young Adulthood
Key Moment
Surgery
Panel Discussion
Monitoring
Caregiver/ Family
Lead Specialist
Emotional/ Psychosocial
Advocacy Support
Information
Patient Impact

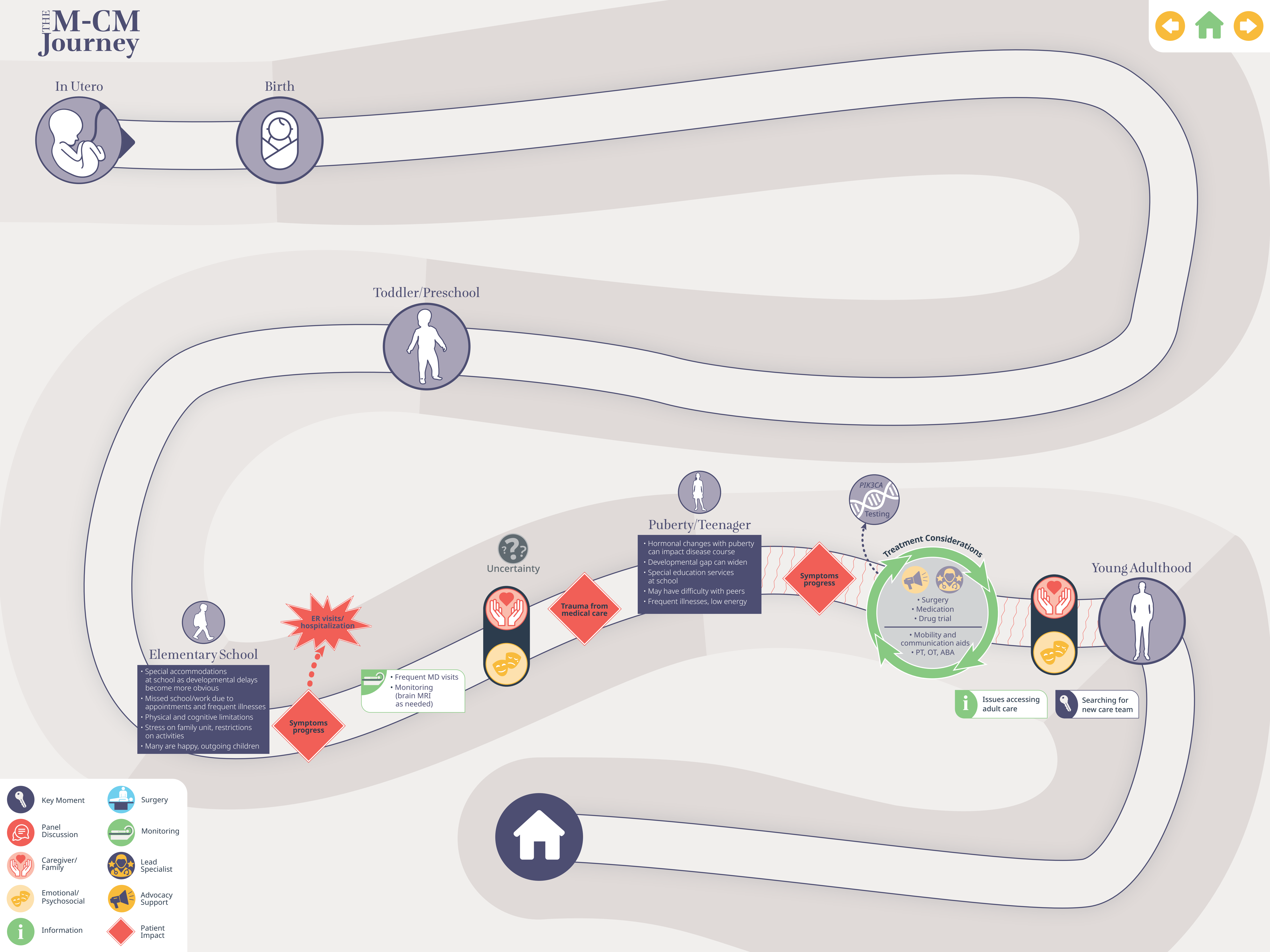
THE M-CM Journey
In Utero
Birth
Toddler/Preschool
Elementary School
• Special accommodations at school as developmental delays become more obvious
• Missed school/work due to appointments and frequent illnesses
• Physical and cognitive limitations
• Stress on family unit, restrictions on activities
• Many are happy, outgoing children
Symptoms progress
ER visits/ hospitalization
• Frequent MD visits
• Monitoring (brain MRI as needed)
Uncertainty
Trauma from medical care
Puberty/Teenager
• Hormonal changes with puberty can impact disease course
• Developmental gap can widen
• Special education services at school
• May have difficulty with peers
• Frequent illnesses, low energy
Symptoms progress
PIK3CA
Testing
Treatment Considerations
• Surgery
• Medication
• Drug trial
• Mobility and communication aids
• PT, OT, ABA
Young Adulthood
Issues accessing adult care
Searching for new care team
Key Moment
Panel Discussion
Caregiver/ Family
Emotional/ Psychosocial
Information
Surgery
Monitoring
Lead Specialist
Advocacy Support
Patient Impact

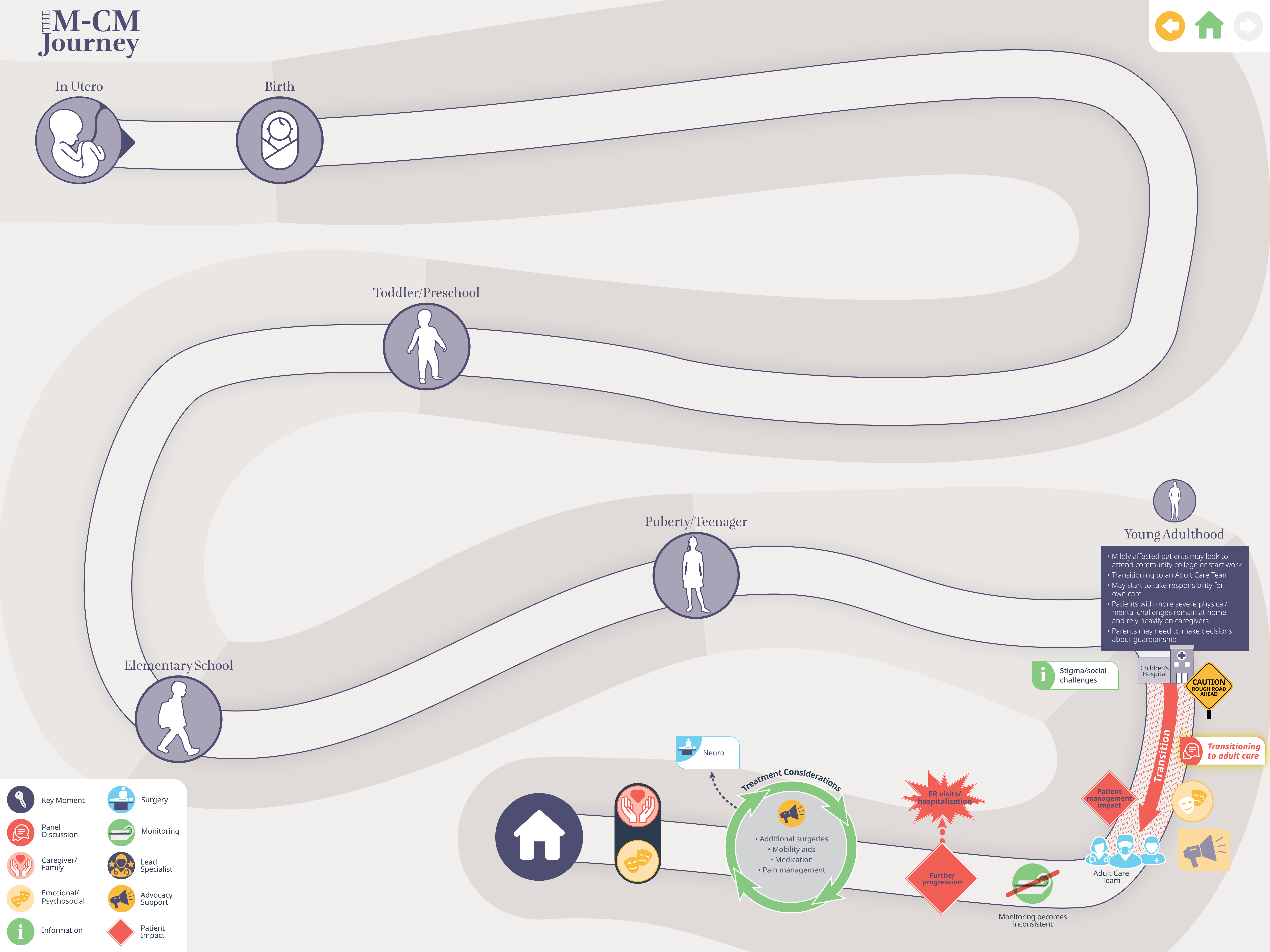

THE M-CM Journey
In Utero
Birth
Toddler/Preschool
Puberty/Teenager
Elementary School
Young Adulthood
• Mildly affected patients may look to attend community college or start work
• Transitioning to an Adult Care Team
• May start to take responsibility for own care
• Patients with more severe physical/ mental challenges remain at home and rely heavily on caregivers
• Parents may need to make decisions about guardianship
Children's Hospital
CAUTION ROUGH ROAD AHEAD
Stigma/social challenges
Transition
Transitioning to adult care
Patient management impact
Adult Care Team
ER visits/ hospitalization
Further progression
Monitoring becomes inconsistent
Treatment Considerations
• Additional surgeries
• Mobility aids
• Medication
• Pain management
Neuro
Key Moment
Panel Discussion
Caregiver/ Family
Emotional/ Psychosocial
Information
Surgery
Monitoring
Lead Specialist
Advocacy Support
Patient Impact
